# Identification of *Vitis vinifera* MYB transcription factors and their response against grapevine berry inner necrosis virus

**DOI:** 10.1186/s12870-023-04296-7

**Published:** 2023-05-26

**Authors:** Xianyou Wang, Shanshan Zhao, Ruijin Zhou, Yunli Liu, Longlong Guo, Huiling Hu

**Affiliations:** 1 School of Horticulture Landscape Architecture, Henan Institute of Science and Technology, Henan, 453003 P. R. China; 2 Henan Province Engineering Research Center of Horticultural Plant Resource Utilization and Germplasm Enhancement, Xinxiang, China; 3 School of Food Science, Henan Institute of Science and Technology, Henan, 453003 P. R. China

**Keywords:** MYB transcription factor, Expression pattern analysis, Biotic stress, Grapevine berry inner necrosis virus

## Abstract

**Background:**

The myeloblastosis (MYB) superfamily is the largest transcription factor family in plants that play diverse roles during stress responses. However, the biotic stress-responsive MYB transcription factors of the grapevine have not been systematically studied. In China, grapevine berries are often infected with the grapevine berry inner necrosis virus (GINV), which eventually reduces the nutritional quality and commodity value.

**Results:**

The present study identified and characterized 265 *VvMYB* or *VvMYB*-related genes of the “Crimson seedless” grapevine. Based on DNA-binding domain analysis, these VvMYB proteins were classified into four subfamilies, including MYB-related, 2R-MYB, 3R-MYB, and 4R-MYB. Phylogenetic analysis divided the MYB transcription factors into 26 subgroups. Overexpression of *VvMYB58* suppressed GINV abundance in the grapevine. Further qPCR indicated that among 41 randomly selected *VvMYB* genes, 12 were induced during GINV infection, while 28 were downregulated. These findings suggest that *VvMYB* genes actively regulate defense response in the grapevine.

**Conclusion:**

A deeper understanding of the MYB TFs engaged in GINV defense response will help devise better management strategies. The present study also provides a foundation for further research on the functions of the MYB transcription factors.

**Supplementary Information:**

The online version contains supplementary material available at 10.1186/s12870-023-04296-7.

## Background

The MYB transcription factors (TF) form the largest family of TFs in all eukaryotes and the second largest TF superfamily in flowering plants [1–5]. The first *MYB* gene identified was the V-myb avian myeloblastosis viral oncogene homolog. Currently, they were found widely distributed in higher plants and play multiple roles in various processes associated with plant growth and development [2–4]. MYB proteins are characterized by a highly conserved N-terminal DNA-binding domain repeat (R), consisting of at least 1–4 imperfect tandem repeats (R) of 50–53 amino acids [1, 3]. In addition, this highly conserved domain comprises regularly spread triplet tryptophan residues that group together to make a hydrophobic core; sometimes, aromatic or hydrophobic amino acid residues replace the tryptophan residues [6]. The N-terminal DNA-binding domains mainly associated with DNA-binding and protein–protein interactions [7, 8]. The MYB domain is highly conserved among high plants, according to the MYB repeat number and the identified of the MYB repeats, MYB proteins are generally classified as MYB-related, 2R-MYB (R2R3-MYB), 3R-MYB (R1R2R3-MYB), 4R-MYB [3, 9]. In plants, the *MYB* gene was first identified in *Zea mays* in 1987 as involved in anthocyanin biosynthesis in aleurone tissues [10]. Subsequently, research has identified the *MYB* gene family members in numerous plant genomes, including 199 in *Arabidopsis thaliana* [11], 492 in upland cotton [12], 299 in cassava [13], 244 in soybean [14], and 192 in *Populus* [15]. Plant MYB proteins play crucial roles in various biological processes, including growth and development [16], cell metabolism [17, 18], cell fate, and stress responses [19]. Recently, numerous researchers have found that MYB TFs play vital roles in signal transduction, hormone synthesis, primary and secondary metabolism, and against pathogen infection [20–22]. In pepper, MYB TF positively regulates the host defense against *Ralstonia solanacearum* infection [23]. In rice, the MYB TF participates in broad-spectrum blast resistance [24]. In apple, MdMYB30 plays a vital role in regulating cuticular wax accumulation and enhances *Botryosphaeria dothidea* resistance [25]. Meanwhile, MdMYB73 regulates the salicylic acid pathway and confers resistance against the fungal pathogen *Botryosphaeria dothidea* [26]. In the Chinese wild grapevine (*Vitis davidii*), MYB TF activates the expression of the *stilbene synthase* gene and positively regulates the defense response against invading pathogens [22]. These earlier findings indicated the role of MYB TFs in modulating plant resistance against pathogens and the underlying mechanisms; however, little is known about the role of MYB TFs in regulating defense responses against plant viruses.

Grapevine (*Vitis vinifera* L.) is among the earliest domesticated fruit species and the most economically important fruit crop worldwide [27]. More than 71 different viruses infect grapes, eventually reducing nutritional quality and commodity value, resulting in heavy losses [28]. Grapevine berry inner necrosis virus (GINV) is a major pathogen that causes severe damage; it belongs to the genus *Trichovirus* and the family Betaflexiviridae. GINV was first identified in 1984 in Japan. Recently, a new variant of GINV has been identified from the “Beta” and “Thompson seedless” grapevines in China [29]. The GINV is widely distributed in China and is highly associated with the ring spot symptom [30]. The GINV-infected grapevine plantlets exhibit leaf chlorotic mottling and ringspot, and the infected berries show discoloration on the fruit surface and necrosis of the flesh [29, 31]. In China, over a third of grapevine plants have been infected with the GINV [30]. GINV infections are closely related to ring spot symptoms. In the field, GINV is transmitted by grapevine erineum mites [29–31]. Therefore, there is the need to identify host factors associated with defensive responses in grapevine plants. The former research results indicated that MYB proteins regulates the host defense against invading pathogens [22–26]. In grapevine, VvMYB TFs were identified only in R2R3 subfamily [32, 33]. In this study, we undertook a comprehensive genome-wide characterization and expression analysis of four distinct subfamilies MYB TFs during GINV infection. The study's findings will provide a valuable resource for subsequent research on the functions and regulatory mechanisms of VvMYB proteins, potentially crucial for antiviral defense response in the grapevine.

## Materials and methods

### Plant materials and growth conditions

The *Vitis vinifera* cultivar “Crimson seedless” and *Nicotiana benthamiana (N. benthamiana)* were used for this study. Berries were collected from 10-year-old grapevine trees grown in a grapevine orchard at the Henan Institute of Science and Technology, Xinxiang City, Henan Province, China (E 113°55', N 35°18'). All grapevine plants were assessed for viruses twice annually, and those tested negative for GINV were chosen for the study. The grapevine berries collected from the same tree were immediately infiltrated with agrobacterium (*Agrobacterium tumefaciens*) cells. The tissues and organs (roots, phloem, leaf blades, fruits) also collected from the same tree of “Crimson seedless” grapevines using tissue-specific expression analysis. We cloned GFP into GINV infection clone plasmid after CaMV 35S promoter using seamless cloning and assembly kit (Mei5 Biotechnology, Co., Ltd, China). Then pGINV-GFP plasmid was transferred into the agrobacterium strain GV3101. *N. benthamiana* was grown under controlled conditions at 25 °C with a 16 h light/8 h dark regime in illumination incubator.

### Identification and sequence analysis of the *MYB* genes

All annotated grapevine proteins were downloaded from the Ensembl Plants database (https://plants.ensembl.org/Vitis_vinifera/Info/Index) and the National Centre for Biotechnology Information database (http://www.ncbi.nlm.nih.gov/) to identify the complete list of grapevine *MYB* genes. *Arabidopsis thaliana* MYB protein sequences were also downloaded from Ensembl Plants and NCBI. A Hidden Markov Model search (HMMsearch) was conducted using the HMM profile of the MYB-like DNA-binding domain (PF00249), which was obtained using the Pfam program (https://pfam.xfam.org/). The preliminary screening of members was performed using HMMER3.1, with *P* < 0.001. All the searched sequences were then submitted to the SMART tool (http://smart.embl.de/) and NCBI CDD (https://www.ncbi.nlm.nih.gov/Structure/bwrpsb/bwrpsb.cgi) to detect the MYB-like DNA-binding domain and to remove the sequences without this domain.

The amino acid sequences of the VvMYBs were analyzed with ExPASy ProtParam (http://www.expasy.org/tools/protparam.html) to obtain the number of amino acids, theoretical isoelectric point (pI), molecular weight, and instability index. Furthermore, *VvMYB* candidate genes were examined using domain analysis programs of Pfam and SMART.

### Chromosomal location and phylogenetic analysis of VvMYBs

All *VvMYB* genes were mapped onto the grapevine chromosomes based on the information available in Ensembl Plants and NCBI databases. The annotation data, including information on the gene's position on the chromosome, chromosome number, and length, were uploaded in the TXT format file. Finally, the genes were mapped to the chromosomes using MapChart software (version 2.3).

A phylogenetic tree was constructed using the amino acid sequences of the MYB proteins of Arabidopsis and *V. vinifera* in MEGA 6.0 software (http://www.megasoftware.net/) following the neighbor-joining (NJ) method (Parameter setting: Bootstrap method-1000 replicates, Poisson model, Pairwisedeletion). We used the trimAl software to remove the redundant sequences information, and used the FigTree software to embellish phylogenetic tree.

### Agrobacterium-mediated infection, RNA extraction, and cDNA synthesis

The agrobacterium (with infectious clone of GINV-GFP, pGD-VvMYB58-Flag, pGD-VvMYB11-Flag, or pGD-GUS recombinant plasmid) were independently transformed into the grapevine berries. The agrobacterium cell with infiltration buffer (10 mM MES/NaOH pH 5.6, 10 mM MgCl_2_, 150 μM acetosyringone) was injected into the “Crimson seedless” grapevine berries with a 1 mL syringe, using mock cells in the infiltration buffer as control. Agrobacterium-mediated transformation method as described in a previous work [34]. Grapevine berries were collected 3, 5, 7, and 9 days after infection. Relative expression levels of coat protein (*CP*) and *VvMYB58* genes were examined using qRT-PCR. Meanwhile, agrobacterium-mediated transient expression of the proteins (VvMYB20, VvMYB58, VvMYB100, VvMYB170, VvMYB191, VvMYB205, VvMYB251, VvMYB258, and VvMYB263) were carried out in *N. benthamiana* leaves, respectively as described previously [35]. The grapevine berries and the *N. benthamiana* leaves were ground individually in liquid nitrogen. Total RNA was isolated from the samples using the Quick RNA Isolation Kit (Waryong, RNAzol, China) according to the manufacturer’s instructions and reverse transcribed into complementary DNA (cDNA) using random pentadecamer primers according to a previously described method [34].

### RT-PCR and quantitative real-time PCR (qRT-PCR)

RT-PCR was performed to analyze the expression levels of *VvMYB* genes (*VvMYB1*-*VvMYB265*), using primers (150–500 bp amplicon) listed in Supplementary Table S5 and the fast PCR mix (Mei5bio, HiPer Taq HiFi PCR mix, China) on a thermal cycler (MyCycler; Bio-Rad). Total RNA was independently extracted from different tissues and organs (roots, phloem, leaf blades, fruits) using Quick RNA Isolation Kit (Waryong, RNAzol, China). The program used was as follows: 3 min at 95 °C, followed by 32 cycles of 95 °C for 25 s, 55–64 °C for 25 s, and 72 °C for 30 s, and a final extension for 5 min at 72 °C. Grapevine *β-actin* gene was used as a housekeeping gene to normalize the cDNA concentrations. Each PCR was replicated (*n* = 3) using the cDNA samples obtained from independent experiments. Agarose gel (1.5%) electrophoresis was used to check the amplicons (150–500 bp).qRT-PCR was performed with the SYBR® PrimeScript™ RT-PCR Kit (Takara) according to the manufacturer’s instructions on an ABI 7500 thermocycler (Applied Biosystems, USA). Grapevine berries were collected at 1, 3, 5, 7, and 9 days after agroinfiltrated. Total RNA was independently extracted from frozen sample also using Quick RNA Isolation Kit (Waryong, RNAzol, China).The relative expression levels of the genes were determined using the comparative ΔΔCT method. Grapevine *β-actin* gene was used as a reference gene.

### Subcellular localization of VvMYB proteins

The full-length coding sequences of *VvMYB20*, *VvMYB58*, *VvMYB100*, *VvMYB170*, *VvMYB191*, *VvMYB205*, *VvMYB251*, *VvMYB258*, and *VvMYB263* without a stop codon were amplified from the cDNA obtained from “Crimson seedless” grapevine berries using gene-specific primers. The fragments were identified by sequencing and fused to the green fluorescent protein (GFP) under the control of the double CaMV35S promoter in the modified plant expression vector pCam35s-GFP to produce pVvMYB20-GFP, pVvMYB58-GFP, pVvMYB100-GFP, pVvMYB170-GFP, pVvMYB191-GFP, pVvMYB205-GFP, pVvMYB251-GFP, pVvMYB258-GFP, and pVvMYB263-GFP plasmids, respectively. All recombinant plasmids were independently transferred into the agrobacterium strain GV3101 and infiltrated in *N. benthamiana* leaves as described in a previous work [35]. Fluorescence signals in the cells were visualized 72 h post-agroinfiltration using an Olympus FluoView 3000 confocal microscope equipped with an Olympus FluoView FV10-ASW 4.0 Viewer Software. Fluorescence images were captured at an excitation wavelength of 488 nm.

### Protein extraction and Western blotting

Protein extraction and Western blotting were performed as described previously [35]. Grapevine berries were ground in liquid nitrogen and mixed with a 2 × SDS sample buffer containing 10% (v/v) β-mercaptoethanol. Proteins were separated by SDS-PAGE (12%), and Western blotting was performed by probing with the rabbit anti-Flag or anti-GFP antiserum (diluted 1:5000).

## Results

### Identification and characterization of the *MYB* gene family in *V. vinifera*

With reference to Ensembl Plants, and transcriptome databases [34], members of the VvMYB family were searched using HMMsearch with a HMM profile of PF00249.

After SMART tool and NCBI CDD verification, a total of 265 *VvMYB* or *VvMYB*-related genes were isolated in the grapevine as candidate *VvMYB* genes across these two databases. We named these sequences according to the corresponding gene identifiers in the genome browsers (Table S1). The gene identifiers (e.g. VIT_00s0194g00130) assigned to genes may help to avoid the confusion that often results when multiple names are used for the same gene in such a large gene family [9, 13]. We distinguished each of the VvMYBs using a provisional nomenclature system and named them from VvMYB1 to VvMYB265 (Table S1). The chemical and physical properties of these VvMYB proteins are shown in Table S1. The length of the *VvMYB* genes varied from 165 bp (VvMYB4) to 9075 bp (VvMYB103) (Table S1). Sequence analysis showed that these VvMYB proteins or polypeptides had a length ranging from 54 to 1738 amino acids (aa), with a predicted molecular weight ranging from 6.27 to 190.21 kDa (Table S1) and a theoretical isoelectric point (pI) ranging from 3.85 (VvMYB27) to 10.79 (VvMYB75) (Table S1). The grand average of hydropathicity index (GRAVY) of the VvMYB proteins was negative (− 1.371 to − 0.149), except for seven MYBs (VvMYB34, VvMYB46, VvMYB154, VvMYB155, VvMYB201, VvMYB202, VvMYB259), indicating hydrophilicity of proteins (Table S1). The aliphatic index of these proteins ranged from 36.4 (VvMYB97) to 123.75 (VvMYB155) (Table S1), while the instability index was more than 40, indicating an unstable protein structure [36]. These observations showed that most VvMYB proteins were unstable, except for twenty-nine (Table S1).

### Classification of VvMYB transcription factors

To verify the reliability of our results, we also performed SMART analysis to identify all of the putative VvMYB protein sequences in Ensembl Plants, and transcriptome databases. The results were consistent with the Pfam outcome. The VvMYB proteins were classified into four sub-families are based on the number and location of MYB-like DNA-binding domain (PF00249). Our study classified the *VvMYB* or *VvMYB*-related genes into four distinct subfamilies MYB-related, 2R-MYB (R2R3-MYB), 3R-MYB (R1R2R3-MYB), 4R-MYB (Table 1, Table S2), consistent with the member grouping in Arabidopsis [9], cassava [13], and apple [37], but there are differences in the quantity distribution. Among them, the R2R3-MYB subfamily accounted for more than one-third of the total VvMYB proteins, while R1-MYB and R1R2R3-MYB subgroups accounted for 29.43% and 26.42%, respectively; the R1R2R3R4-MYB subgroup accounted for 6.04% of VvMYBs only (Table 1).

**Table 1:** VvMYB transcription factor types distributed on grapevine chromosomes

Chromosome	R1-MYB	R2R3-MYB	3R-MYB	4R-MYB	Total
Vv_chr1	9	9	7	0	25
Vv_chr2	6	8	5	1	20
Vv_chr3	6	4	0	0	10
Vv_chr4	4	6	5	2	17
Vv_chr5	2	2	5	1	10
Vv_chr6	1	8	3	0	12
Vv_chr7	9	2	4	1	16
Vv_chr8	5	6	7	1	19
Vv_chr9	3	4	0	0	7
Vv_chr10	3	0	0	1	4
Vv_chr11	3	5	4	0	12
Vv_chr12	4	4	3	0	11
Vv_chr13	4	3	3	2	12
Vv_chr14	2	10	11	2	25
Vv_chr15	5	4	1	1	11
Vv_chr16	3	5	4	1	13
Vv_chr17	2	9	3	3	17
Vv_chr18	5	10	1	0	16
Vv_chr19	2	2	4	0	8
Total	78	101	70	16	265
Percentage	29.43%	38.11%	26.42%	6.04%	100%

### Chromosomal distribution of *VvMYBs*

**Fig. 1:**
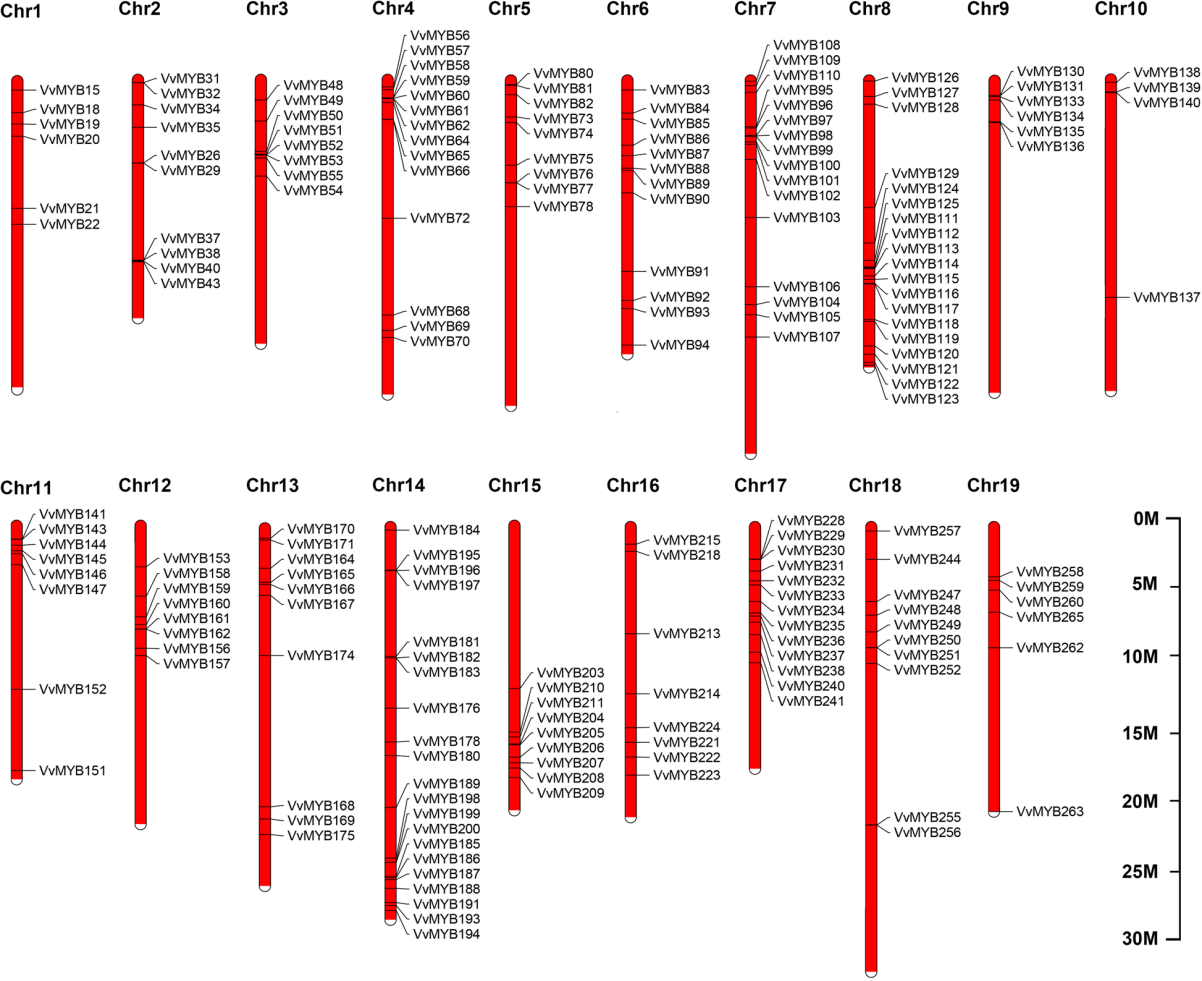
Distribution of *VvMYB* genes on grapevine chromosomes. The chromosomal position of each *VvMYB* was used to map them onto the grapevine genome

Analysis of the chromosomal distribution of 265 possible members of the *VvMYB* or *VvMYB*-related genes based on Ensembl Plants and NCBI annotation. Genome chromosomal location analysis revealed that 209 *VvMYB* genes were unevenly distributed on all 19 chromosomes (Fig. 1). The TFs *VvMYB1* to *VvMYB13* were distributed on scaffolds (Chr0). Meanwhile, the chromosomal location information was unavailable for forty-three of the *VvMYBs*. We also found that the density and distribution pattern of the *VvMYB*s on the chromosomes were not uniform. Some chromosomal regions and single chromosomes (chromosomes 4, 7, 8, 11, 14, and 17) showed a high density of *VvMYB*s, while others did not (chromosomes 1, 10, 16, and 19) (Fig. 1, Table S3). The highest density of *VvMYB* genes was observed on chromosomes 8 and 14, with 19 and 21 *VvMYB* genes, and the lowest density was observed on chromosome 10, with only 4 *VvMYB* genes (Fig. 1, Table S3).

### Phylogenetic analysis of *VvMYBs*

**Fig. 2:**
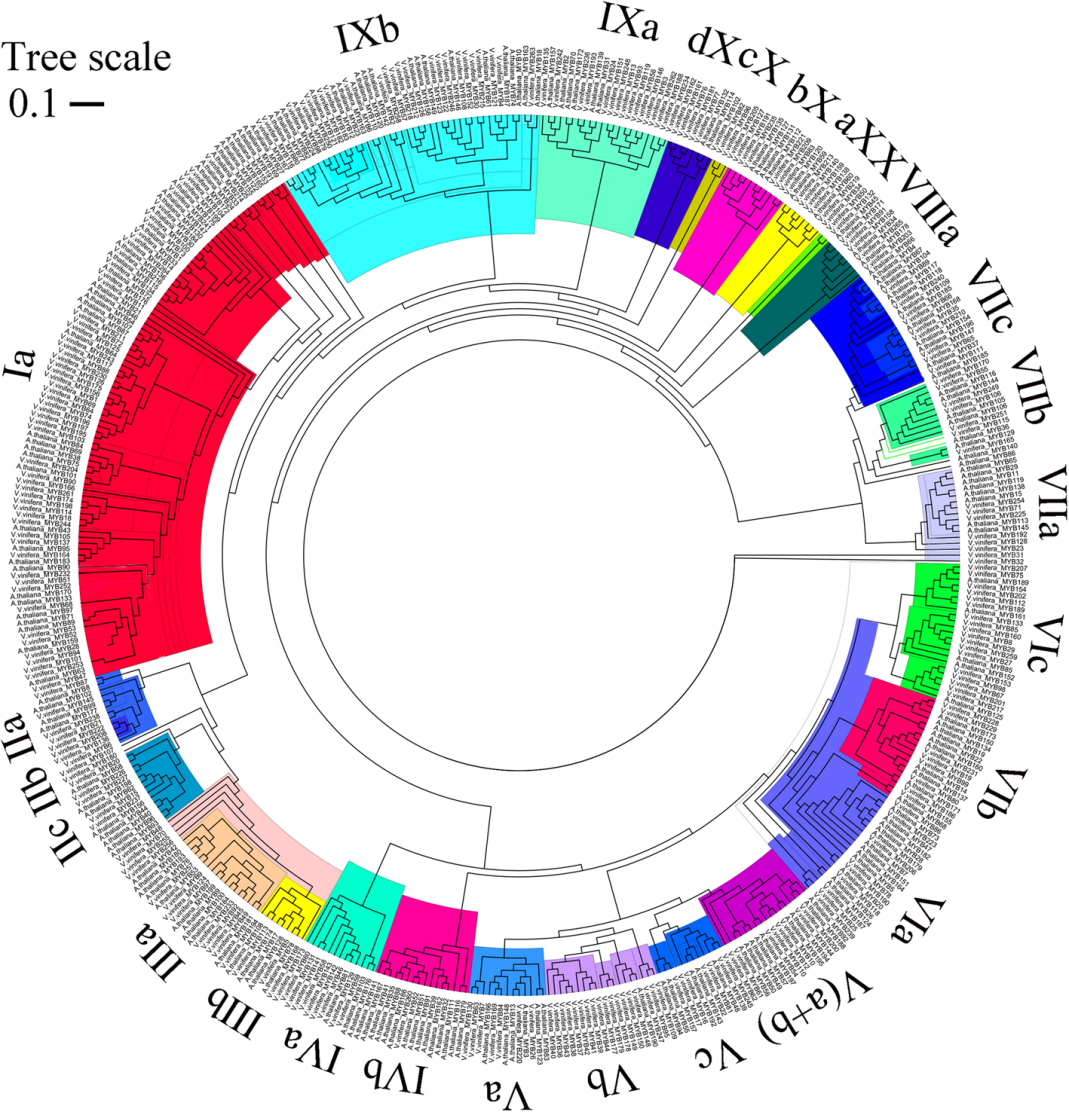
Phylogenetic tree of 265 grapevine MYB proteins and 199 Arabidopsis MYB proteins. The phylogenetic tree was constructed with MEGA 6.0 using the neighbor-joining method. The bootstrap test was performed with 1000 iterations. All the subgroups are shown in different colors

Furthermore, to elucidate the potential evolutionary relationships between the various VvMYB TFs, we constructed an NJ phylogenetic tree using the amino acid sequences of the grapevine and Arabidopsis MYBs. We used the amino acid sequences of 199 *AtMYB* genes (AtMYB1 to AtMYB199) obtained from the Ensembl Plants and NCBI database for the analysis. The AtMYB grouping was consistent with the provisional nomenclature of the VvMYB subfamilies. Analysis of the phylogeny and the protein sequences categorized the 199 AtMYB genes and 265 VvMYB, respectively. As shown in Fig. 2, 265 VvMYB proteins were distributed among 26 subfamilies, which were designated Ia through Xd (Fig. 2). Most MYB subgroups contained more grapevine members than Arabidopsis [12]. The largest subfamily was Ia, with 56 VvMYB proteins, and the smallest was X, with only two VvMYB proteins (Fig. 2, Table S4). The VvMYB domain is highly conserved as other species, according to the number of MYB-like DNA-binding domain (PF00249).

### Tissue-specific expression profiles of *VvMYB* genes

**Fig. 3:**
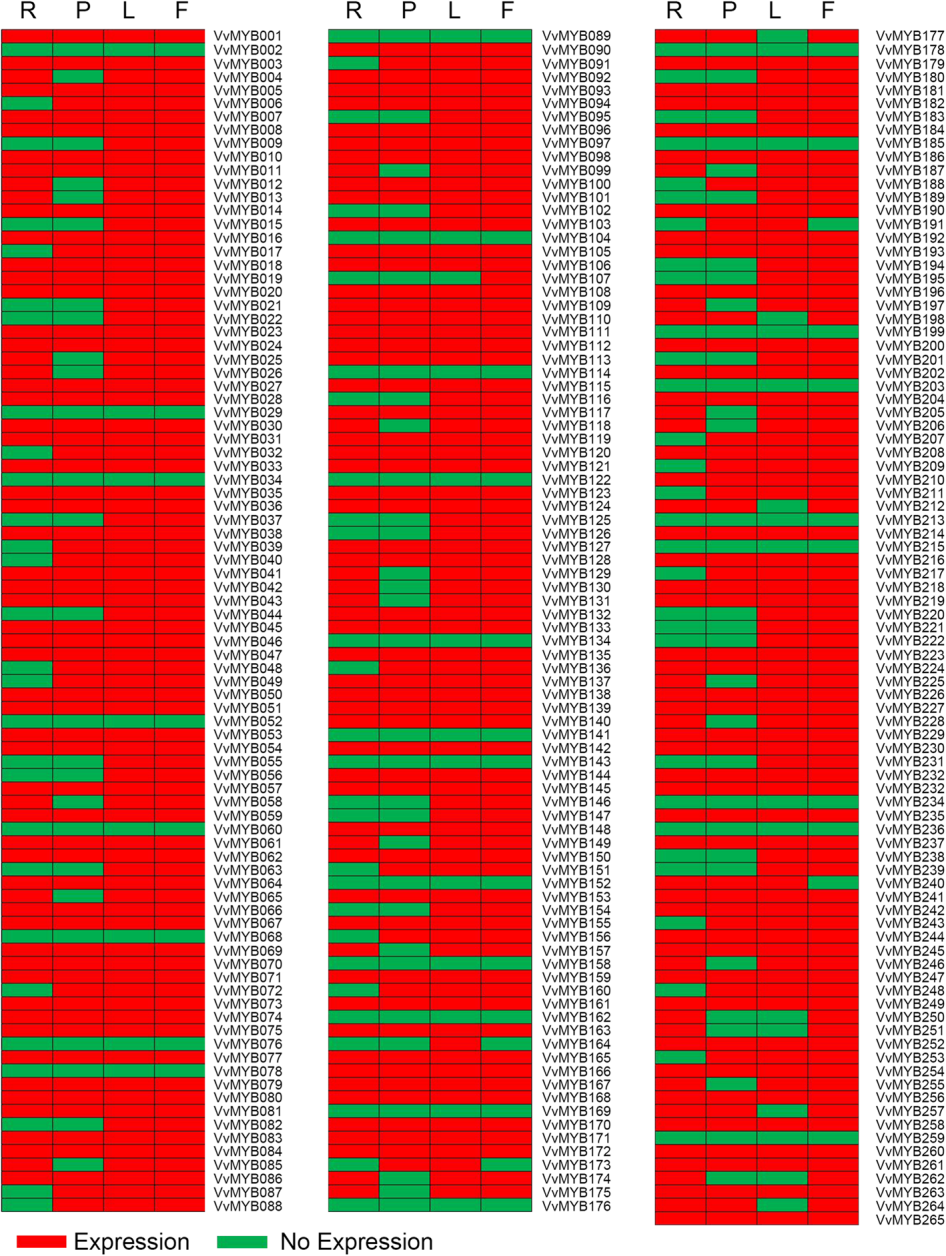
Tissue-specific expression patterns of *VvMYB* genes in “Crimson seedless” grapevine. R, roots; P, phloem; L, leaf blades; F, fruits (berries). The Heat maps are based on the RT-PCR expression profiles analyzed data using MeV software

Generally, the *MYB* genes exhibit different expression patterns under biotic or abiotic stress responses and physiological and developmental stimuli. Studies have also demonstrated the variable expression of *MYB* or *MYB-*related genes in different varieties or tissues or under various developmental phases [13]. In this study, we performed a RT-PCR to investigate the expression profiles of *VvMYB* genes in roots, phloem, leaf blades, and fruits (berries) of grapevine. Among the 265 *VvMYB* or *VvMYB*-related genes, 236 *VvMYB* genes exhibited various expression patterns in different grapevine tissues. However, we did not detect any transcripts of 29 *VvMYB* genes (11.33%) in these four grapevine tissues. Thirty- *VvMYB* genes (11.32%) were expressed in two tissues (leaf blade and fruit), and most *VvMYB* genes were expressed in the leaf blade (85.66%) and fruit tissues (87.55%) of the grapevine (Fig. 3, S1).

### Subcellular localization analysis of VvMYBs

**Fig. 4:**
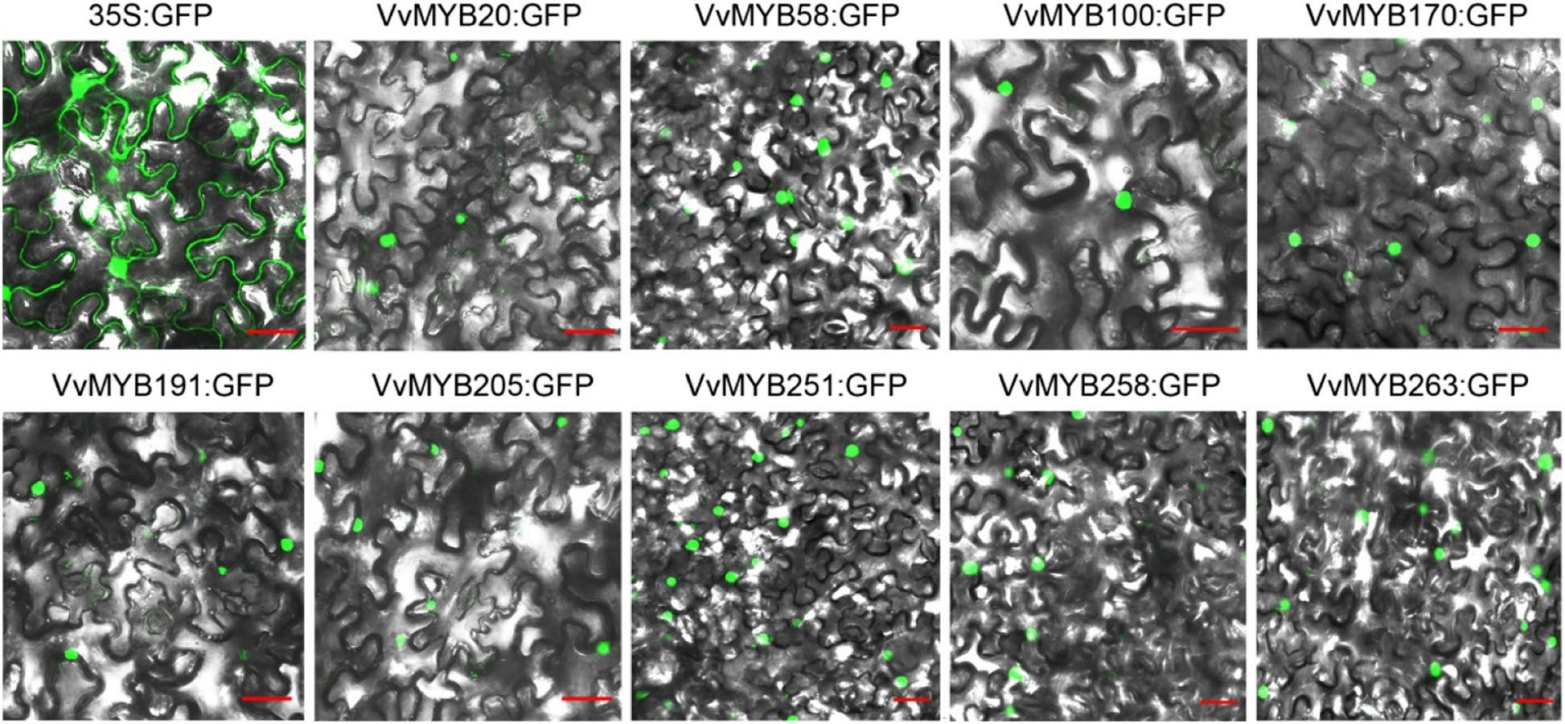
Subcellular localization of VvMYB proteins in *N. benthamiana* leaf. VvMYB20, VvMYB58, VvMYB100, VvMYB170, VvMYB191, VvMYB205, VvMYB251, VvMYB258, and VvMYB263 are shown. Bars = 20 μm. Each assay was repeated three times

Most TFs are localized in the nucleus. To investigate the subcellular localization of VvMYB proteins, we randomly selected nine *VvMYB* genes using transient expression in *N. benthamiana*. We cloned the full-length cDNA of *VvMYB20* (NM_001281204.1), *VvMYB58* (XM_010650081.2), *VvMYB100* (XM_002280991.3), *VvMYB170* (XM_002272670.4), *VvMYB191* (XM_002283539.3), *VvMYB205* (NM_001281231.1), *VvMYB251* (XM_002284201.3), *VvMYB258* (XM_002284212.4), and *VvMYB263* (XM_010646850.2) from “Crimson seedless” grapevine by RT-PCR and fused to the C-terminal GFP fusion protein to analyze their subcellular localization. Nine VvMYB:GFP fusion proteins and pCam35s-GFP vector separately transferred into the agrobacterium strain GV3101 and infiltrated in *N. benthamiana* leaves. We observed GFP fluorescence of nine fusion proteins in transiently transformed *N. benthamiana* leaf epidermis at 3 days post-inoculation (dpi) only in the nucleus. Among them, three fusion proteins (VvMYB58:GFP, VvMYB251:GFP, and VvMYB263:GFP) were highly expressed in *N. benthamiana* leaf (Fig. 4).

### Expression analysis of *VvMYBs* genes in response to GINV infection

**Fig. 5:**
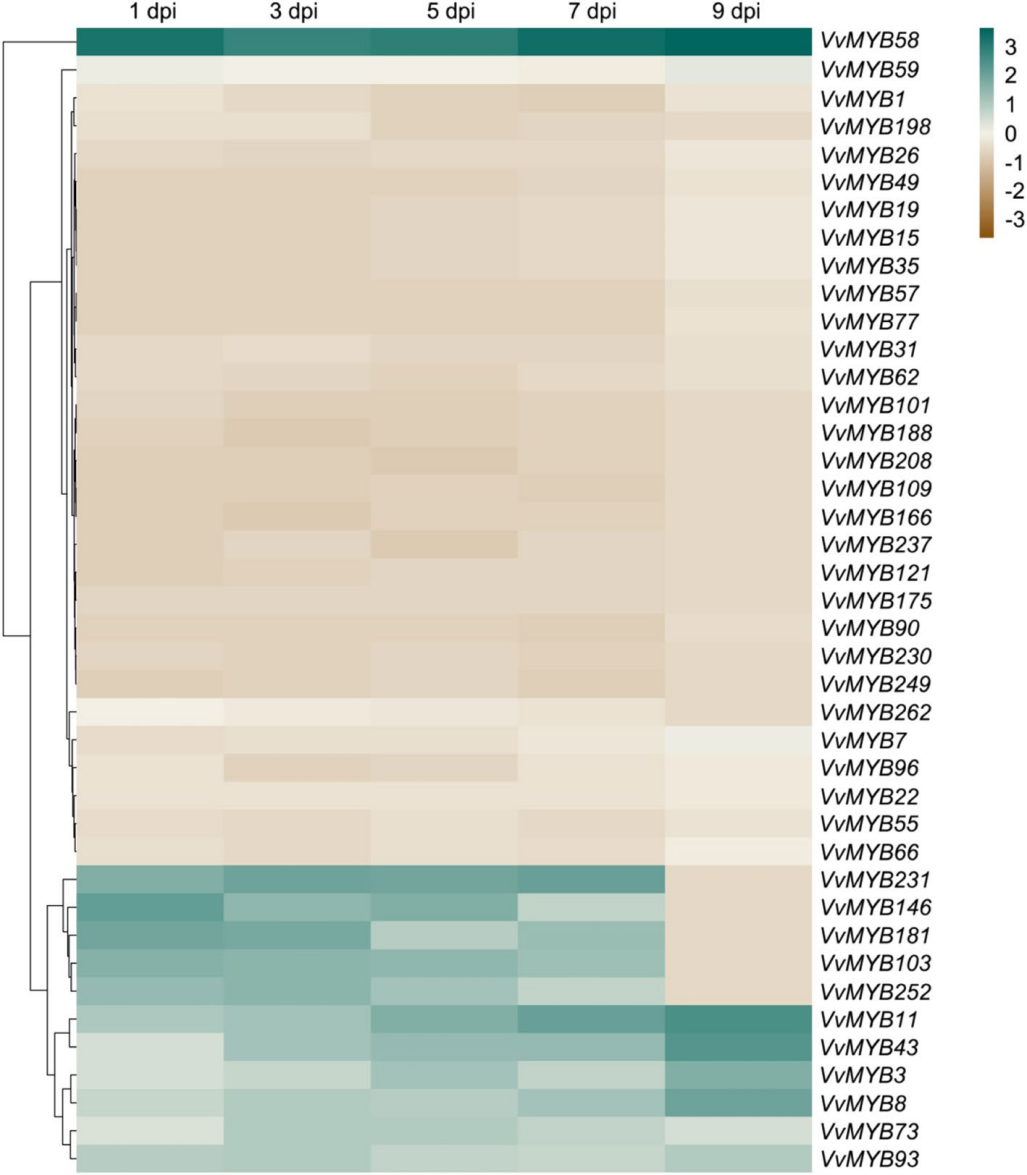
Expression profiles of randomly selected *VvMYB* genes during GINV infection. The expression of *VvMYB* genes in “Crimson seedless” grapevine during GINV infection was analyzed using qRT-PCR. The inoculated grapevine berries were harvested at 1, 3, 5, 7, and 9 days post-infiltration (dpi)

Further, to examine the role of *VvMYB* or *VvMYB*-related genes in grapevine under GINV infection, we compared 41 *VvMYB* genes expression between GINV infection and mock using qRT-PCR. These were selected based on the transcriptome data sets [34]. In previous studies, we only selected one time point (7 dpi) using transcriptome sequencing. To investigate their expression profile in grapevine berries at various time intervals, we selected five time points at 1, 3, 5, 7, and 9 dpi. Here, 12 *VvMYB* genes were upregulated, while 28 *VvMYB* genes were downregulated during GINV infection; *VvMYB59* showed no significant difference during the entire process (Fig. 5, Table S6). Among them, *VvMYB11* and *VvMYB58* were markedly induced at five time points during GINV infection. *VvMYB11* and *VvMYB58* were continually induced during GINV infection (Fig. 5, Table S6). Fourteen *VvMYB* genes were significantly downregulated during GINV infection. These genes mainly distributed in three subfamily VII, VIII, and X (3/14, 4/14, 3/14) (Figs. 2 and 5). Taken together, these results demonstrated that *VvMYB* genes actively regulate defense response against GINV in the grapevine.

### VvMYB58 suppresses GINV accumulation

**Fig. 6:**
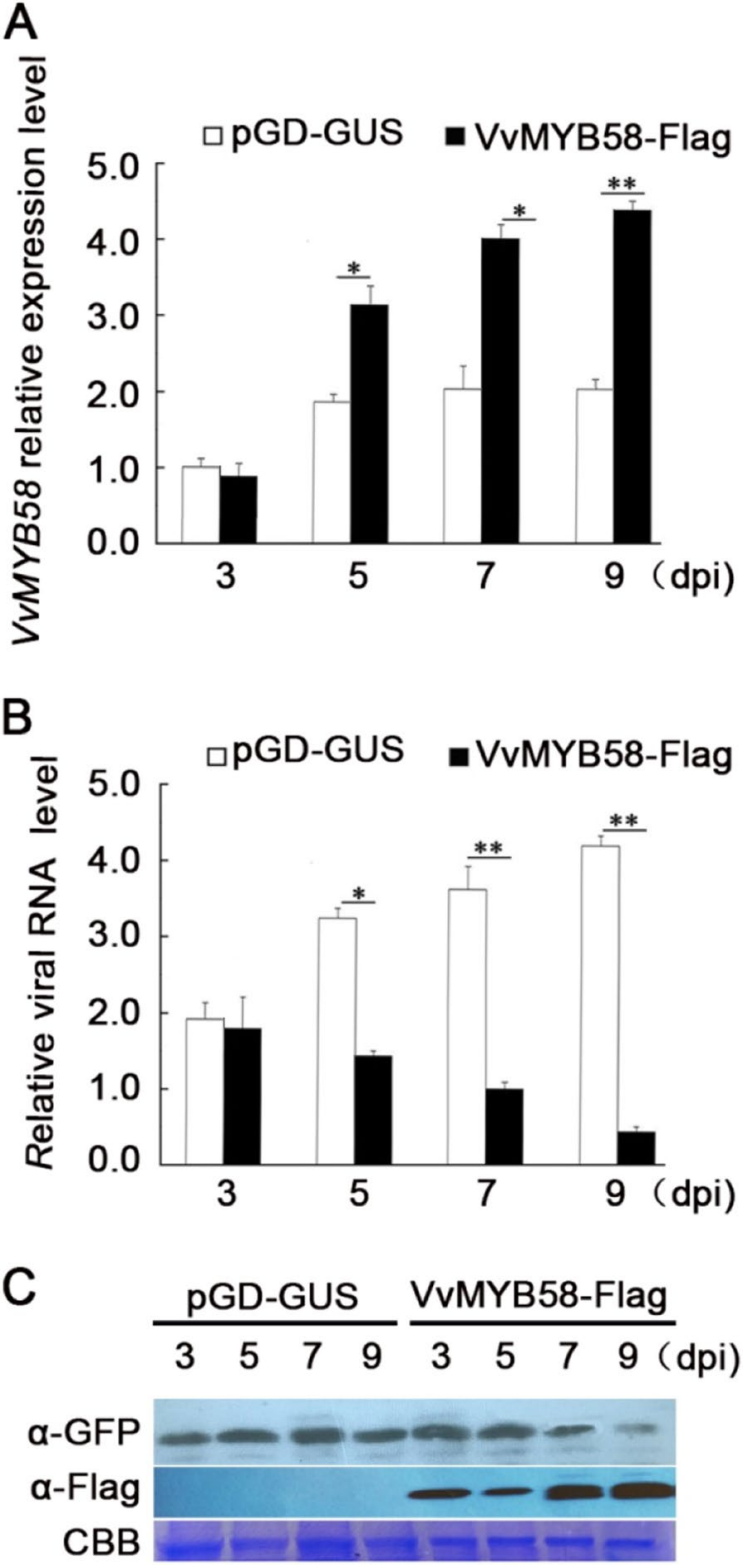
Overexpression of VvMYB58 reduces GINV RNA abundance. (A) and (B) pGD-VvMYB58-Flag and GINV-GFP were co-infiltrated into grapevine berries by Agrobacterium-mediated transient transformation; pGD-GUS and GINV-GFP served as the negative control. Error bars represent the SD of three independent biological replicates. *P < 0.05, **P < 0.01. (C) GINV-GFP and VvMYB58-Flag protein accumulation levels. These proteins were detected using anti-GFP antiserum and anti-Flag antibodies, respectively. CBB, Coomassie blue staining confirmed equal sample loading

GINV infection significantly increased the expression of mainly *VvMYB11* and *VvMYB58* (Fig. 5, Table S6). Therefore, to investigate whether *VvMYB11* or *VvMYB58* is involved in defense responses in grapevine, we cloned the full-length cDNA of *VvMYB11* (XM_002264114.4) and *VvMYB58* (XM_010650081.2) by RT-PCR from “Crimson seedless” grapevine. We independently cloned the two genes into pGD-Flag vector, and generated pGD-VvMYB11-Flag and pGD-VvMYB58-Flag recombinant plasmids. The agrobacterium with infectious clone of GINV-GFP and pGD-VvMYB58-Flag were co-infiltrated into the grapevine berries. And GINV-GFP and pGD-GUS were co-infiltrated served as a negative control. qRT-PCR assays indicated that the transcript levels of VvMYB58 at 5, 7, and 9 dpi were 326%, 413% and 478% of that in controls (Fig. 6A), and the relative levels of GINV RNA were about 43%, 26%, and 16% of that in controls (Fig. 6B). Western blot analysis showed that transient expression of VvMYB58-Flag leads to an obvious decrease in viral accumulation (Fig. 6C). “Crimson seedless” berries infiltrated with GINV showed necrotic symptoms at 7 dpi (Fig. S2D), which is consistent with our previous research [34]. Overexpression of VvMYB58 suppressed GINV replication but does not affect symptoms (Fig. S2E). In addition, the GINV agroinoculated *N. benthamiana* plants behaved chlorotic mottle at 7 dpi (Fig. S2E). Nevertheless, overexpression of VvMYB11 did not affect GINV accumulation (Fig. S2A-B). The results demonstrated that the overexpression of *VvMYB58* decrease GINV abundance in the grapevine berries (Fig. 6).

## Discussion

Since the initial identification in 1982 [1], the MYB family of TFs has been a research topic. Currently, the *MYB* genes have been evaluated in about 74 species with sequenced genomes [38], such as Arabidopsis [9], upland cotton [12], cassava [13], soybean [14], apple [37], and populus [15]. Over the past few decades, plant MYBs have emerged as key regulators of responses to diverse abiotic stresses, such as salinity stress [39], high- and low-temperature stress [40], drought [41], and phosphate starvation [42]. The MYB TFs also play crucial roles in regulating the responses against infection by pathogens, such as bacteria [23, 24] and fungi [25, 26]. However, little is known about the MYB-mediated transcriptional regulation during grapevine response to viruses. The previous research have been identified *VvMYB* genes only in R2R3 subfamily, which explored wine quality-related MYB [32] and regulation of stilbene accumulation [33]. This study conducted a comprehensive genome-wide analysis of the *VvMYB* gene superfamily in the grapevine plants. The study identified 265 *VvMYB* or *VvMYB*-related genes from the grapevine plant genome and transcriptome database [34]. These findings were to refine and expand the member of grapevine MYB superfamily [32, 33]. Our results provide a robust theoretical foundation for further antiviral defense response studies in grapevine.

The present study found similarities in the conserved sequences among the VvMYB proteins within the same subfamilies; however, differences were detected in their chemical and physical characteristics, such as pI (VvMYB75 and VvMYB157 or VvMYB167) and the instability index (VvMYB218 and VvMYB72) (Table S1). These differences may be due to the discrepancies in the amino acids in the non-conserved regions of the VvMYB members, suggesting that each VvMYB protein may act differently in its microenvironment [4, 38]. Further sequence analysis showed that these VvMYB proteins or polypeptides vary widely in length (54 to 1738 amino acids), and most VvMYBs are hydrophilic (Table S1). These chemical and physical properties have the similar characterization to the Arabidopsis MYBs [12, 13]. Analysis of the physiological and biochemical properties suggested similar pI, GRAVY, instability index, and aliphatic index for most VvMYB proteins (Table S1), indicating a close evolutionary relationship. Moreover, the study found an uneven distribution of 209 *VvMYB* genes on the 19 chromosomes of the grapevine (Fig. 1). The *VvMYB* genes appeared concentrated in the homologous regions in chromosomes (Fig. 1). Our results showed ten *VvMYB* genes within a 2–6 Mb region in Chr4, eleven *VvMYB* genes within a 3–10 Mb region in Chr7, sixteen *VvMYB* genes within a 10–23 Mb region in Chr8, eleven *VvMYB* genes within 20–28 Mb region in Chr14, and thirteen *VvMYB* genes within a 4–13 Mb region in Chr17 (Fig. 1). These results suggest that the segmental and tandem duplications contributed to the expansion of *VvMYB* genes in the grapevine. The MYB domain is classified as VvMYB-related, R2, R3, or R4 type based on the number of repeats [43, 44]. Similarly, the present study classified the 265 *VvMYB* or *VvMYB*-related genes into four distinct subfamilies (Table 1, Table S2). However, our observations on the distribution are not simlar to the reports by Salih et al*.* on upland cotton MYB [12]. Grapevine has an almost similar number of VvMYB R1, R2, and R3 members, but most upland cotton MYBs belong to the R2-MYB subfamily, and differences in the number of R2R3-MYB subfamily members, reflecting species differences (Table 1). We identified 26 subgroups containing 265 *VvMYB* genes (Fig. 2). The largest subfamily VIIIa, only had seven VvMYB proteins. Meanwhile, the VvMYBs were widely distributed in Ic, III(a + b), and VIIIb subfamilies, and Ic and VIIIb groups had two main branches (Fig. 2). The VvMYBs were not clustered into the AtMYB subfamilies VIIIa and Ib. These results indicated that the VvMYBs and the AtMYBs separated during the evolutionary process.

In plants, MYB TFs are involved in various processes, such as cell fate and identity [38, 45], primary and secondary metabolism [46], development [47], and biotic and abiotic stress responses [48]. The complex function of the MYB protein family is reflected in the tissue-specific expression patterns of the various members. Nearly half of VvMYB TFs exhibited differences in the expression patterns in grapevine plants (Fig. 3, S1), similar to cotton [12] and cassava MYBs [13]. Regulation of the specific stress genes temporal and spatial expression patterns is essential to plant stress responses [49]. Studies have confirmed the role of MYB TFs of Arabidopsis in defense responses [3]. In Arabidopsis, *Botrytis cinerea* induced the expression of *BOTRYTIS SUSCEPTIBLE1* gene encodes the R2R3-type MYB TF, which increased tolerance to necrotrophic pathogens and abiotic stresses [50]. Meanwhile, the R2R3-type MYB30 of Arabidopsis positively regulated programmed cell death associated with hypersensitive response [51]. However, there is little research on the functions of MYB TFs in defense responses in the grapevine. Therefore, the present study analyzed the temporal and spatial expression patterns of *VvMYBs* during GINV infection to understand their roles in defense responses in the grapevine. The qRT-PCR results indicated that among the 41 randomly selected *VvMYB* genes, 12 were induced during GINV infection, while 28 were downregulated (Fig. 5, Table S6), indicating an alteration in *VvMYBs* during GINV infection. Subsequently, to verify whether *VvMYB* family members are involved in antiviral defense responses in grapevine, we overexpressed *VvMYB11* and *VvMYB58*, which were significantly induced during GINV infection (Fig. 5). Our results demonstrated that the overexpression of *VvMYB58* suppressed GINV abundance in grapevine (Fig. 6), confirming its role in antiviral defense response in grapevine. Moreover, in present study overexpression of *VvMYB11* gene did not directly affect GINV-enriched in grapevine. However, *VvMYB11* was dramatically induced during GINV infection (Fig. 5). VvMYB11 TF may not works alone in regulation antiviral defense response gene in grapevine. This result showed that differences in functions among the various members of VvMYB. Taken together, our results indicate part of *VvMYB* gene family members play an crucial role in protecting against GINV infection. *VvMYB58* was functionally characterized as a target gene for genetic engineering approaches to improve the disease resistance of fruit trees and other crops to multiple biotic stresses. Thus, a model was proposed to aid in understanding of the *VvMYB* genes regulate defense response against GINV in grapevine.

## Conclusion

The present study performed the first comprehensive and systematic analysis of the *MYB* gene superfamily in the grapevine. The study identified 265 *VvMYB* or *VvMYB*-related genes and divided them into 26 subfamilies according to their evolutionary characteristics. Additionally, our results revealed that *VvMYB* genes were distributed across the entire grapevine genome. In grapevine berries, 12 *VvMYBs* were induced, while 28 were downregulated during GINV infection. This means that part of MYB TFs play vital roles in against GINV infection. In the subsequent research, we will deeply explore the molecular mechanism about *VvMYB58* restricts GINV enrichment. And *VvMYB58* whether also suppresses other viruses or pathogens. Simultaneously, we now have rich resources for subsequent studies of gene cloning and functional characterization of members in this VvMYB family.

## Supplementary Information

**Additional file 1: Table S1.** Summary information of physiological and biochemical properties of the VvMYB proteins.

**Additional file 2: Table S2.** The VvMYB transcription factors distributed on different subgroups.

**Additional file 3: Table S3.** Distribution of VvMYB genes on grapevine chromosomes.

**Additional file 4: Table S4.** Summary information of VvMYBs and AtMYBs amino acid sequence FASTA file.

**Additional file 5: Table S5.** The primers sequences used in this study.

**Additional file 6: Table S6.** Expression patterns of 41 VvMYB genes during the process of GINV infection by qRT-PCR.

**Additional file 7: Fig. S1.** Tissue-specific expression patterns of VvMYB genes in “Crimson seedless” grapevine. R, roots; P, phloem; L, leaf blades; F, fruit.

**Additional file 8: Fig. S2.** Overexpression of VvMYB11 did not affect viral replication. (A) and (B) pGD-VvMYB11-Flag and GINV-GFP were co-infiltrated into grapevine berries by Agrobacterium-mediated transient transformation; pGD-GUS and GINV-GFP served as the negative control. Error bars represent the SD of three independent biological replicates. *P < 0.05, **P < 0.01. Overexpression of VvMYB58 did not affect the symptoms of virus-infected. Comparison of symptoms of GINV virus-infected GINV virus-infected Nicotiana benthamiana (C) and “Crimson seedless” grapevine fruits with mock at 7 dpi (E). (D) shows symptoms of solitary infection GINV of “Crimson seedless” grapevine fruits.

**Additional file 9: Fig. S3. **The original image blots in Fig. 6C.

**Additional file 10: Fig. S4.** Part of the original image of Fig. 3.

## Data Availability

All annotated grapevine gene sequences were downloaded from the Ensembl Plants database (https://plants.ensembl.org/Vitis_vinifera/Info/Index) and the National Centre for Biotechnology Information database (http://www.ncbi.nlm.nih.gov/). All relevant data presented in this study are provided either in the manuscript or additional files.
