# Supplementary material for: Identification of Vitis vinifera MYB transcription factors and their response against grapevine berry inner necrosis virus
Source: BMC Plant Biol. 2023 May 26;23:279. doi: 10.1186/s12870-023-04296-7 (PMC10214588; doi:10.1186/s12870-023-04296-7)
Supplement: Supplementary file 5 — Additional file 5: Table S5. The primers sequences used in this study. [file 12870_2023_4296_MOESM5_ESM.docx]

**Table S5** The primers sequences used in this study.

| Gene | Forward primers (5´→3´) | Reverse primers (5´→3´) |
| --- | --- | --- |
| *VvMYB1* | TGGAATTCAAGGAACCTGA | GAGCTCCCTGACCGCTTGTA |
| *VvMYB2* | GTCAATCCGGAGCTTCTAAG | CTTGAGGAAGGCGTCGATAG |
| *VvMYB3* | CTAGCAGCTTGGGATCT | CTAATTCAAGGTCTGGATAT |
| *VvMYB4* | ATGGTGAGGACTCCGTACTA | CTTAGCTTCAACAGTGGATGT |
| *VvMYB5* | GACCAATGGAACCGATGTCA | GTGGCTAACTTCAGAACTCT |
| *VvMYB6* | CTAGCACTCCATCATCTGTT | GAAATATTGTAGTGCATCTTCA |
| *VvMYB7* | AGCTAGGGACCTCCAGAGAC | TGTCTGGATGATGTCTAAGT |
| *VvMYB8* | CGGCAATGGTGGAGAGGTTG | GACGATAGCTTCTTCTAGC |
| *VvMYB9* | ATGTCATATGATCCAACAGTG | GCAGTTGAGTTCCTTGGCGC |
| *VvMYB10* | ACGTGGACCATTGCGCATTG | CTCAATGTACATAGCTATAG |
| *VvMYB11* | CCTAAGCTTGCCGGACTCC | TCGAGTAACCATGCACAACT |
| *VvMYB12* | ATGGCGCACAATACTTATGG | TGGTCCACGTGAAATTGCAA |
| *VvMYB13* | AGATTGGCCTTAAGCGATGT | GAGGCAAACA TGTTCATGGA |
| *VvMYB14* | TCAAGAGAGGGCTATGGTC | TGCTAAAATGGACATGAAAT |
| *VvMYB15* | ATATGAGCCTTCGCCTGATT | CACAGGATCAATTAACACCT |
| *VvMYB16*  *VvMYB17* | GGAAGAGTTGCAGGTTAAGA  TGGAGAAGAGAACTGGAAGC | CTTCCCAATGTCTCAGAATG  CTACAGTATCTTCCTCTTCC |
| *VvMYB18* | ACGATGAAGAAGTCTTAACC | CTCGACCTTCTTATCACACT |
| *VvMYB19* | TTGCTTGCCATGGTGAAG | AGGAATAGTTGAGCTCTGGG |
| *VvMYB20* | TAAGCAAGATACCAACAAAG | GTTCAAACCAGATGATG |
| *VvMYB21* | ATGTCTCGTTCCTGCTCTCA | TGTAACTGAGGCGGAGAGAC |
| *VvMYB22* | TAATACTGACGCTCCTCCTA | CCATTGTTAAATAAAGCCCT |
| *VvMYB23* | ATGGTCTCAGCTGTTACTCA | TAGGGTTCTCTTCATTTATG |
| *VvMYB24* | CTACATTGAGGAACACGGCC | CCATGTGGCTGAGATTTG |
| *VvMYB25* | ATGAAAGGAGAGGATAACTA | CCCACATCCATATTATTATC |
| *VvMYB26* | TAGATGAAGAAAGCAGACCA | CGTAGCTTCCGATCTCTTCT |
| *VvMYB27* | ACTGCAATTTGAGGACTACT | TCATGTATACATATCCTCC |
| *VvMYB28* | ATGGAAGTTGAAGGTGAAGT | TCATGTATACATATCCTCC |
| *VvMYB29* | GCAGTTTGAGGACTACTT | GCTTCTTCATCTGGTTCATG |
| *VvMYB30* | AATGGGTCTGTCTTGGATTG | GAATCCAACTTCCCACCACA |
| *VvMYB31* | GGTTCCGAGAAATGGGTCTG | GAATCCAACTTCCCACCACA |
| *VvMYB32* | AATGGGTCTGTCTTGGATTG | GAATCCAACTTCCCACCACA |
| *VvMYB33* | ATGGTTGTTGGAGTGAAGTC | TGATTCACCATGTCCAGTTG |
| *VvMYB34* | GTGGACATCAGAAGAAGAAG | AGCCTCCAATATGTGACTGT |
| *VvMYB35* | TAGATGAAGAAAGCAGACCA | CGTAGCTTCCGATCTCTTCT |
| *VvMYB36* | TCCACTCCCTCCTCTTCTTC | TGCCCACTATCGGTTTCCTT |
| *VvMYB37* | TAGATGAAGAAAGCAGACCA | GATCTCTTCTCCACTCTCGC |
| *VvMYB38* | GCTCAATGTCCTCCCGCAGC | AGGGCACTTTGTCTGAGTCG |
| *VvMYB39* | ATGGAGAGTTTGGGAGTTAG | CCCTAGAAAGAGTTTGCAAG |
| *VvMYB40* | ATGGAGAGCTTAGGAGTTAG | TTGTGGTGAAGTGGATGATG |
| *VvMYB41* | TGGACTCGGGAAGAAGATAT | AAGCGATGATGGTGCCAGTA |
| *VvMYB42* | TAGTAAGGAGGGAATGAATA | TCTGATGGAGGAGAAGTTAT |
| *VvMYB43* | GCTGCTCAGAGATTATGTTA | TTCTGTCGTCTTTGCTTTGG |
| *VvMYB44* | AGCTGTAGATTGAGATGGTT | TTCAATGGTATGGTCAGCGA |
| *VvMYB45* | TTGTTCAAGCTTCTAGTATC | ATCTCTCTCAACAACACCTG |
| *VvMYB46* | TACTCACGGCTTACATTAAA | CTTGTGGCCTTGGTTCGGAT |
| *VvMYB47* | ATGGCGAGAAGTCCTTGTTG | ACTGGTTCGGACTGGCATGC |
| *VvMYB48* | GCCTCATCGCTTATATCCGG | AGATATGGTTGTAACGTCCG |
| *VvMYB49* | CCTCGACGATGTGCTTCCTG | CACCGATGCCAGACTTCTAT |
| *VvMYB50* | CGTGACTGGATTATAAAGAG | ATCAGCTGAGCAGGAGTTAC |
| *VvMYB51* | TCTGGCTCATCCTGGACTGC | GGTAGTCTTGTAATTGGGGA |
| *VvMYB52* | ATGGCATCAGGGTCTCGCAT | TTATACACTTCAGCAGCCTC |
| *VvMYB53* | ATGGCATCAACCTCTCTCAA | AGTTGGGAATAGGAACACGG |
| *VvMYB54* | GGAATGAATAAAGGAGCCTG | GAGAAGTTATCATGATGGGA |
| *VvMYB55* | ATGGAGGCTTTGCAGAACGA | CGATACAGATTTGGAGAAGA |
| *VvMYB56* | AACGTGAAGAGAGGGCCATG | GGAGCCGAGCTGAAAGGATT |
| *VvMYB57* | CTGTGGGAACATTGGTCATA | TGACTGGCTACTTGGGTTGG |
| *VvMYB58* | GAAGATAAGATACTCACGGC | TAGTTCTTGATTTCATTGTC |
| *VvMYB59* | TAGTAAGGAGGGAATGAATA | TAGTTCTTGATTTCATTGTC |
| *VvMYB60* | AAGGAAAGTGGAGGAACATT | TCTTTAGACTCATGATTTGG |
| *VvMYB61* | ATCACTCTTCAAACCCTGCT | CGTTATCTGTTCTGCCTGGA |
| *VvMYB62* | GCTGCTCAGAGATTATGTTA | GGTGCTTCATCCATGTTAGA |
| *VvMYB63* | ATGGCTTCTACTATCAGTCA | TTCTTGATCTCATTGTCTGT |
| *VvMYB64* | CAATCCTCTGAAGCGAAGTT | GACTTTGCACTCCATTCATC |
| *VvMYB65* | ATCAGCTCCACCTTCATCTT | CTTATGGGTTCTCGGATCTA |
| *VvMYB66* | CCGGAGCAGGAAGTGGTTGT | CCGCAACTTTCTTTGGAGTC |
| *VvMYB67* | GGTTTATGACGGAGCTCTGT | ACCTTCTGGAACTTGTCATC |
| *VvMYB68* | TAGTAAGGAGGGAATGAATA | TAGTTCTTGATTTCATTGTC |
| *VvMYB69* | AAGTGGTTGTGACGGAGCAG | TCTTACTCCGGAAAGCTTGT |
| *VvMYB70* | CTATGGACAAAGGAGGAGGA | TTCTTCTTCTTGATGCCGAG |
| *VvMYB71* | CGATGGAGGATTCTGCCGGC | GCGAGCTATCAGAGACCACT |
| *VvMYB72* | CCTTTCTATCAGCTGGTAAT | TGGTCTGGGAGGAGGAATCT |
| *VvMYB73* | ATGGGTGCTGGAGCTCTGTC | TTGTGGGTGTTTGGGTCGAT |
| *VvMYB74* | TAGTAAGGAGGGAATGAATA | GGTGTTCCAGTAGTTCTTGA |
| *VvMYB75* | TCTATCTCCGTCACTTGTTG | TTTACAGAAGCAGACTTAGT |
| *VvMYB76* | ATGGCGAGAAGTCCTTGTTG | ACGCTGCTGCAGTAGAGTGG |
| *VvMYB77* | ATGGTAAGAGCTCCTTGTTG | GTATCCTCTTCTTCTCTGCT |
| *VvMYB78* | TCATAGAAGAAGGGTGGAGG | AGACTTGTCGGAAGGGAGTT |
| *VvMYB79* | ACGCCTGTGTTGGTTGATAA | CTCAGGATTCTTCGGTACAG |
| *VvMYB80* | ATGGATGTTAATGGTAGAGG | GAGAGTGATGTTTCCACGTC |
| *VvMYB81* | GCCATTCATCCAACCACAAG | GGCGAGCATCCTGAACTACC |
| *VvMYB82* | AGGGAAGATGGACAGAGCAA | GGAGGAGGGTATGGATTCAG |
| *VvMYB83* | CATCCTCAGCATCAGCTCCA | CTTATGGGTTCTCGGATCTA |
| *VvMYB84* | ATGAGGAAGCCTGAGAAGAG | TGAGCCCTCCCATGACATCC |
| *VvMYB85* | AGAGCAGAAACTAGACACAC | ACTCCTTCAGTTCCTTCCAG |
| *VvMYB86* | AGGGAAATCAACAGAAGAAT | GATTCCCAGCACATTCTCCT |
| *VvMYB87* | ATGGTGTTGCAGAAGAGGTT | TATAACAGAAGCAAGTGCTG |
| *VvMYB88* | TTTCCAACTCCAATTGCCTG | CTTCAACCCTTCATATCCAT |
| *VvMYB89* | ATGGCGGGTGGTGGTGGTGG | GCCACCTTCTCTTCTCCGCT |
| *VvMYB90* | TCTCTGGTCAGACGTCTCAT | GCAGCCTCAATGTCATCCCT |
| *VvMYB91* | ATGGCCGGTGATCAAGAAGG | TCGATTATGCGGTGCTCCTC |
| *VvMYB92* | TCTATCCAGCTTCGCATATA | GAACCATAGTGGCGGTCCTC |
| *VvMYB93* | TGACAAGGCAAATGTGAAGA | TGGTGGTAGTTTCCTCTGAG |
| *VvMYB94* | CGCCCTCTCCTTCTTCCTCC | TTCCGCTCCGACTCGCTCTG |
| *VvMYB95* | CGCCTCACCCACCTTACTTC | TTCGTCACCGTCGCCGATCC |
| *VvMYB96* | ATGAGGAAGCCTGAGAAGAG | CTAGGCTGATCAGATGACGA |
| *VvMYB97* | GAATGATGGCAGGGCACTTG | AGACTTGTCGGAAGGGAGTT |
| *VvMYB98* | CCTGCAGTGGACGTCTCTCA | AATGAAGCGGAGATCAAATC |
| *VvMYB99* | CTCCGGCCGACTTCTAATGA | GCCCAAACGAACTCCTCCAT |
| *VvMYB100* | AAGTATGGCCATGGCTGCTG | AGGGTTGCTGTCTTCTCTAT |
| *VvMYB101* | ATGGCCGGTGATCAAGAAGG | GCTGCAGTCTCTTGATCCGG |
| *VvMYB102* | AAGGTCCATGGACTCCCGAA | GGGACGCATCAAGAGAGTGT |
| *VvMYB103* | AGAGTTCATTTGTCCAGCCA | TTCTCCCGACTTCATGTGAA |
| *VvMYB104* | AATGCAGACACGGACACCTC | GTACATGGTCCACAGCGGCT |
| *VvMYB105* | TGAATCTTCCTTCTGCACTA | ATGTTCTTGTACAGTCCATG |
| *VvMYB106* | GGTAGCAGCGATGATGGGAG | ACTGCACTGCCTCAACCTTT |
| *VvMYB107* | TGAGAAGGAACACACCAACA | TCTTGCTAGTGCTGTTGCTT |
| *VvMYB108* | CTCCTTGTTGTGATGAGAGT | CAAACATATCAGTTCGAGGC |
| *VvMYB109* | CAGAACCAGAGGCGGCCGAG | CTCACCTTCACGAGCTTCCC |
| *VvMYB110* | AGAGGACACTGGAGGCCTGC | CACAGCGTTGTCGGTTCTAC |
| *VvMYB111* | ATGGCTGATGTGAAACTGGA | TTGTTTCCCTATGCGACCTG |
| *VvMYB112* | ATGAAGGAGAGGCAGCGTTG | TAGAATCCGATCGTACTTGC |
| *VvMYB113* | ATGGTGTTGCAGAAGAGGTT | TGGCAGATCAGAATTAATCG |
| *VvMYB114* | ATGACCAAGATCGACGCTCT | TTCAAGGAGGAGAGAGGGCT |
| *VvMYB115* | ATGGAGGATATAACAGTGGT | CATTATCTGTTCTCCCTTGA |
| *VvMYB116* | ATGAGGAAACCTTGCTGTGA | GTCAGTCCTTCCTGGCAATC |
| *VvMYB117* | TCTCTCTCCGGCCTCATATC | CTCTTCTTCGGTCCACGGCA |
| *VvMYB118* | GCTCAATGTCCTCCCGCAGC | AGGGCACTTTGTCTGAGTCG |
| *VvMYB119* | AATGTGAAGAAAGGCCCATG | CTGTTGTTGTTACCGCATGT |
| *VvMYB120* | CTCCGGCCGACTTCTAATGA | TATCCAATCTTTCTGCCATT |
| *VvMYB121* | GAAATGATTGCCTTGAGATG | CACTTAGGTAAACATGCTCT |
| *VvMYB122* | ATGAAGCAAGAGCAACTCAC | ACCAGAGTAGACGTCGAAGT |
| *VvMYB123* | ATGACAATGATGGGAGTGAT | GCTCTTCTCTGGACTTCCGT |
| *VvMYB124* | TTGAAGTTGAGCATTTACTT | GAGAGACTATTTGTTGCATG |
| *VvMYB125* | GCTTCTCCGCCACCACCACC | TTTGACGAGTTGGTGGGTGG |
| *VvMYB126* | TACAGCAGATGCATCCGATC | ACATCACGTCCACGCATTGT |
| *VvMYB127* | GGTATCAAGAAGGGTCCT | GTACAGGTGCTTGTGGTTGA |
| *VvMYB128* | AAGTGGTGGTGGTGGGAATT | CCTCTCTCAGCTTGGCATCC |
| *VvMYB129* | TGAGCCCTGGGATAGTGA | ACAACCAGTTGGGATCCTAG |
| *VvMYB130* | ATGGCTGAGAAGGAAGTGAA | CTCATTATCTGTTCGACCTG |
| *VvMYB131* | AACGTGAAGAAGGGACCATG | GGCCTGTGAATATGGATTAT |
| *VvMYB132* | GGCCTCAGATCTGTCTTCCA | TTCTCTGCCTTCTTCTTCTT |
| *VvMYB133* | AGTTTGAGGACTGTGTGGTT | CTGCATCTATTTCTGTGTTC |
| *VvMYB134* | CAACAAACAGAAGGTGAAGA | TGATGATCTTGAGGACTCGG |
| *VvMYB135* | ATGTCGGCAATATCAGGTGT | TCAAGAGGCTGGTGCAATGG |
| *VvMYB136* | ATGACTCGTCGCTGCTCACA | CCGGTGCTCCTCTTCTGTCC |
| *VvMYB137* | ATGACCGGAGATGAAGTAAG | CCTGATCTGACCTTGAAGCC |
| *VvMYB138* | CAGAATACTCTACTTCTAAT | TGGTTGAGTATCTTGAATTC |
| *VvMYB139* | GGCCTCAGATCTGTCTTCCA | TATCTTCTTCGCTGCATTCT |
| *VvMYB140* | CCTCAAATGTCACCACTAGT | CAATCATGTTGTTTAGTCGA |
| *VvMYB141* | GAGGACAAGATTCTAACAGA | GATCACATTAGTCTCCGTTT |
| *VvMYB142* | AACCCTACTGTGTGAAAGAG | CGGATGACTGTATGATGAGA |
| *VvMYB143* | AAGCTGAGGAAAGGCCTGTG | TCTTGTCCGTTCTCAGCCTC |
| *VvMYB144* | GAGGAGGAGGAGGCGCTACG | TGTGGCACCTCATGTCTTTG |
| *VvMYB145* | ATAGGCCATAATTCAAGGAC | ACGCCGCTTCTTCTTGCTGA |
| *VvMYB146* | ATGGGTAGAGCTCCTTGTTG | GGGAACTGTACTGAGCTGAG |
| *VvMYB147* | GGAGGACTACTGGCCCTACA | ATGGCAGGATTTAGATGATT |
| *VvMYB148* | ATGACAATGATGGGAGTGAT | GCTCTTCTCTGGACTTCCGT |
| *VvMYB149* | ATGGAGGAGACTGGAGGACT | TGCTCTTCTCTGGACTTCCG |
| *VvMYB150* | ATGGAGGAGACTGGAGGACT | TAGCAATCCTCGGCGTCTCT |
| *VvMYB151* | TGACAAAGCCAACGTGAAGA | AGTTGCATCTTTAGGGTCCT |
| *VvMYB152* | CTCCTTGTTGTGATGAGAGT | TATGAGGTGAGGCAAGCTTG |
| *VvMYB153* | CGATGGTGGTGGCGGTGGCG | GGAGGAGCCTCGATGGAACT |
| *VvMYB154* | AGGGACCATGGACGGCTGAA | CTGTTGTTGAGGTTGGGCAG |
| *VvMYB155* | ATGTCGGCAATATCAGGTGT | ATCAGCAGTTTCAAGAGGCT |
| *VvMYB156* | AAGCTGAGGAAAGGCCTGTG | TCTTGTCCGTTCTCAGCCTC |
| *VvMYB157* | TGACAAAGCCAACGTGAAGA | AGTTGCATCTTTAGGGTCCT |
| *VvMYB158* | ATGAGGAAACCTTGCTGTGA | CGGTTTCCTAAGAGAGCATG |
| *VvMYB159* | ATGGTGTTGAAGAAGAAGCA | TTTGCAAGTTACCATCACAT |
| *VvMYB160* | CGGTGATGGCGGAGAAGCTG | CATAAGCACGCAAAACTCCA |
| *VvMYB161* | AGATATGGGTGTTGGAATTG | AGAGACCATTGGAGATGAAG |
| *VvMYB162* | AGATATGGCCATTGGAACTG | TAGTTCTTGATCTCATTGTC |
| *VvMYB163* | AGCTCAGCCATTATATCCAG | GCTGAGAAGAGATGATATGT |
| *VvMYB164* | TCCATGTCTCCACTATACCT | ACCATCACATCACAACCAAC |
| *VvMYB165* | GTGGGAGCAGTAGCCGAGTC | AGTGGTTCTTGATTGCATTG |
| *VvMYB166* | TCTATCCAGCTTCGCATATA | GGCGGTCCTCTTTCTTAACG |
| *VvMYB167* | TCAAGGAGAGGAAGCACGAG | CATGTTTGGAGATGCATTGC |
| *VvMYB168* | ATGGAGATGGAAATAGAAGA | GAGAGTACAGCTTCAGTGTG |
| *VvMYB169* | ATCACCATTACATCAACCAA | CCTCAGCTTAGTATTCCAGT |
| *VvMYB170* | ATGACTCTGACCACCGGCGG | CGACTCGGACGACACTGTTG |
| *VvMYB171* | CACTGCAGAAGAAGAAAGGC | CTTCTGGCGATGCATTGTTG |
| *VvMYB172* | CTGGCAACTGGAGAACTCTT | ATTGAGTTGGGATGAACTGC |
| *VvMYB173* | GAGGAGCTTGGACTGTTGTG | CTAAATGCCTTGGTCCGAAT |
| *VvMYB174* | TGATAAAGGGAGGCGTGTGG | CATTTCCTTCTCATCCTCAT |
| *VvMYB175* | AGTAACGACGACAACAACAT | GACGTCGTTGTCATTCTCCT |
| *VvMYB176* | AGGGACCATGGACGGCTGAA | TGGTTCTTGATTTCGTTATC |
| *VvMYB177* | GTGAAGAGGATCATCTGCTA | CACTGTCTCCTCAACTTCAT |
| *VvMYB178* | CTAAGTTGTTTATGGACGGC | TTCTTGCTGAGGTGGGTGTT |
| *VvMYB179* | ATGGAATTCCTCTTCTGTGG | GTACTTCTTGCTCTGGTGGG |
| *VvMYB180* | CATGGTGAAGGCTGTTGGCG | CCGGATGACATGGTAGCAGC |
| *VvMYB181* | CTCCTTGTTGTGAGAGTATG | TGCAGTCTTTGATCTTCTTG |
| *VvMYB182* | ATGAGGATAATGATAAAGGG | CATTTCCTTCTCATCCTCAT |
| *VvMYB183* | ATGAGGATAATGATAAAGGG | CATTTCCTTCTCATCCTCAT |
| *VvMYB184* | AAGCTGAGGAAAGGCCTGTG | TCTTGTCCGTTCTCAGCCTC |
| *VvMYB185* | ATACCAGCTCCAAACACAAA | CTTCTTCAGATCAACCTTCT |
| *VvMYB186* | CTGCTGGACTGAAACGTACT | GACTTGGCTTGTAGTTGCTT |
| *VvMYB187* | ATGGAGGAAGTGAATCAGTA | TCGGATCCTTTCTGCATTTC |
| *VvMYB188* | ATGAAGCTCTCTGCAATTGC | CCTCAGCTTAGTATTCCAGT |
| *VvMYB189* | GCAACCAGAAGAAGACGCAC | AGATGGGTGGTCTTGTGGAG |
| *VvMYB190* | ATGGAGGAAGTGAATCAGTA | TTGTCTGTTCGTCCTGGTAC |
| *VvMYB191* | TACTAGTCTCTTATATCCAG | TAGTTCTTGATGTCATTGTC |
| *VvMYB192* | ATGGTACACAAGCGTCCTTT | ACTGCCCTTACTCCATGGTC |
| *VvMYB193* | AACTCTTGGCTTACATTGAA | TCACTGGAGAGTAGAGCATC |
| *VvMYB194* | ATGGTACACAAGCGTCCTTT | ACTGCCCTTACTCCATGGTC |
| *VvMYB195* | AAATCTAGGCAACGGAAGAG | TTTGCAACTGATGGAGGCTG |
| *VvMYB196* | TAGAGGTGTGAATAAGCGGT | ATCTGTCATCATTGCTATTA |
| *VvMYB197* | GAATTCAACCAAATCTAGGC | TTTGCAACTGATGGAGGCTG |
| *VvMYB198* | ATGGCAAACGATGAGATGAT | CATGACTGTGCACTCATCGA |
| *VvMYB199* | ATGGGAAGACAGCCTTGTTG | TTTAGCCGCTTCTTGATTCG |
| *VvMYB200* | ATGTTGGCGAGGCTGAGAGG | GTGGATCCCGATTGAGCC |
| *VvMYB201* | ATGGCAAGAACACAGCAGAA | AGGTGTTCCTCTAGGTAGAG |
| *VvMYB202* | GCTGTGTTTGAGGACTTGTT | CTGAAGCCAAACCTCAGCAT |
| *VvMYB203* | ATGGAAGGAGAGAGAGAGGA | TGGGAAGAAGATGGTGAAGA |
| *VvMYB204* | GCTGTGTTTGAGGACTTGTT | CTGAAGCCAAACCTCAGCAT |
| *VvMYB205* | TTCTAAGGTTGGTTTGCATA | GTTCTTCCTCCTCTTGGGCT |
| *VvMYB206* | CATAAACATGACAGAACAAG | TTGTATCTCTTGAGCTCTTT |
| *VvMYB207* | ATGTCGGCAATATCAGGTGT | CCCACTTATTGCCATGCTGC |
| *VvMYB208* | TAAACCCTAACCCACCACAA | GGAGGAGGCACATGTTCACT |
| *VvMYB209* | AAGAATGGCCATGGAAGCTG | CACTCTCCCATTGCGCCATG |
| *VvMYB210* | ATGTCGGCAATATCAGGTGT | ATCAGCAGTTTCAAGAGGCT |
| *VvMYB211* | ATTATCAAGACAAGGAAACC | CAGGATGCGAAGAAGAAACT |
| *VvMYB212* | ATGGCTGGATCACTGCAGGC | TGGAGTGGTGTCTGAGTGGA |
| *VvMYB213* | ATGTCGGCAATATCAGGTGT | CCCACTTATTGCCATGCTGC |
| *VvMYB214* | ATACCACCACCCAGCGAAGC | GGGCATGGAGATCGATGACC |
| *VvMYB215* | ATGGCGAGATCACCGGAG | GTACTCAAATTGGCTGCTGC |
| *VvMYB216* | ATACCACCACCCAGCGAAGC | TCTCGTTGTCTGTTCTTCCA |
| *VvMYB217* | ATGTCGGCAATATCAGGTGT | TCAAGAGGCTGGTGCAATGG |
| *VvMYB218* | ATGGCAAGAACACAGCAGAA | CACTAGCCTTATCATCCATG |
| *VvMYB219* | ATGAGGAAACCTTGCTGTGA | CTAGGAAAGTTGCATCGTTG |
| *VvMYB220* | TCACCGGAAGAAGATGCTAG | TTAGAGTTGATGCATTGGGA |
| *VvMYB221* | CGATAAGCATGGAGTGAAGA | CCCTAGCGCTCTCCCACTGA |
| *VvMYB222* | ACTTCGATCCCGCTTCAATG | GAGCAGCTGAAGAATGGAAA |
| *VvMYB223* | CATAAACATGAGCGAACAAG | GCATACCCTTGGCGATTTCT |
| *VvMYB224* | ATGGATTCGCAGGTGGAGAC | TCCCGACATTCTCCGCCAAC |
| *VvMYB225* | GAGGCCAGCTGAGGATGAGA | CCGATCATCTGCTGCCTATC |
| *VvMYB226* | CATGGTGAAGGTAGCTGGCG | GTCAGTCCTTCCTGGCAATC |
| *VvMYB227* | AGAGTGGAGAAAGTGATGAC | GGAGTTACTAGCATCTTCAG |
| *VvMYB228* | AGAGTGGAGAAAGTGATGAC | GGAGTTACTAGCATCTTCAG |
| *VvMYB229* | AGAGTGGAGAAAGTGATGAC | AAGGATTCCAAATTCAGAGT |
| *VvMYB230* | CCCTAGTTCTCCGCGGTAC | GTTAGGGCTGAATCGGACGC |
| *VvMYB231* | AAGTTGAGGAAGGGTCTGTG | CTTCGGTTTCATTCAGTGGC |
| *VvMYB232* | ATGGAGCAGAGAGGAAGAGA | TGCTCCTCTTCTGTCCATGG |
| *VvMYB233* | ACTTCGATCCCGCTTCAATG | GAGCAGCTGAAGAATGGAAA |
| *VvMYB234* | AACGTCAAGAAAGGCCCATG | GCCTGTTGTTCTTTGCGCTG |
| *VvMYB235* | GATCATGGTCCAGGGAATTG | TCTCTCCCACTGACCTTTGG |
| *VvMYB236* | CTCCCTGCTGCGACACCACC | TTAGAACGGCATGGCTGGGC |
| *VvMYB237* | TGATGATGAGGAGTTATACG | AATAGTAGTCATCAGAGTCG |
| *VvMYB238* | CTCAGTGTGGTCATAATGGT | TTGAGATTGTTTCTGCGGAG |
| *VvMYB239* | CTGTGGACAGTGGAGGAAGA | ATACTGTGAGCCTCCTTAGC |
| *VvMYB240* | AATTACCTGAAATCTTACAT | CTTGATTTCTTGACAGTTGA |
| *VvMYB241* | AAGTTGAGGAAGGGTCTGTG | ATTCAGTGGCTTGTGGGTGT |
| *VvMYB242* | GAGGATGAGAAACTGTACAA | CTTCGGTTTCATTCAGTGGC |
| *VvMYB243* | ATGTCTCAATTCATAGATTA | GTCGTGATATCATGAATACT |
| *VvMYB244* | ATTCGGACTCATTGGACTCG | AGAACTTCTGGGCATGGCTG |
| *VvMYB245* | ATTGGACTCGCCTTGATGAT | TGGCGGAGGTAGAACTTCTG |
| *VvMYB246* | TGATGAGAATGGTCTGAAGA | TGGTGTGTCATGGGATCAAA |
| *VvMYB247* | GAATGCGTTGGTGAATAAGA | CGGAAACTGGAATAGTGATG |
| *VvMYB248* | AACGTCAAGAAAGGCCCATG | AGCTTCTTCTTCAGTCTCGT |
| *VvMYB249* | GAAGCGTTGCAGAGACTGGT | GTTATAGACGAGCATTTCCG |
| *VvMYB250* | CTCCGGCCGACTTCTAATGA | GCCCAAACGAACTCCTCCAT |
| *VvMYB251* | ACCTCTTCTTCCGATTCTTC | CTCTGCCTCTTCTTCAACGT |
| *VvMYB252* | ATGGGATCGAGTTCAAATAA | GTAGTTAGGCAATGGGACTT |
| *VvMYB253* | ATGCTTTCTCCTCTTCATGT | ATCAACACTTCCTCCGCTGC |
| *VvMYB254* | ATGGAGGAATCTAGTGATGA | TGGAATCACTAAGAACATCT |
| *VvMYB255* | CGACAAAGCCCACACCAACA | GTTTCTTCGGTGGTGATTGG |
| *VvMYB256* | CGACAAAGCCCACACCAACC | TGGTGGTTGGTTTGGTTGGC |
| *VvMYB257* | ATGAGGATGATAGACTTGGG | GTTGCAATACATGGACCAAA |
| *VvMYB258* | AAGGGCCATGGACTGTAGAG | TCCTGATGCGAGTGTTCCAA |
| *VvMYB259* | CTCTGATGGCCCACAAGTTG | TTAATCTGAACATGAGCACA |
| *VvMYB260* | CTCTGATGGCCCACAAGTTG | TGATGATTCTTGAGCTCCTG |
| *VvMYB261* | TTATTCATCATCATCAGCTA | TAAAGTGACTAGGTTCCTCC |
| *VvMYB262* | TTATTCATCATCATCAGCTA | AAACCATGTGTGCTCCTCCT |
| *VvMYB263* | AAGAGTAAGGTGAAGAGAGG | GGGCTCATTGTTAGTACTAG |
| *VvMYB264* | GTTGCAGATTGAGATGGATT | GAGAAGAGATCAGATTGTGA |
| *VvMYB265* | GAAGACCGGCCGGAGAATGG | CTGGTTGACGGTATTGCTCT |
| *Vvβ-actin* | CCCCATGCTATCCTTCG | AGGCAGCTCATAGTTCTTCTC |
| *CP* | ATGCCGGTAATTAGGGAATA | GATGTGATCG CATTAGTCTT |
| pVvMYB20-GFP | GGATCCATGAGGATGCCCTGC | TCTAGATCCAAAGAGGAGCAGCG |
| pVvMYB58-GFP | GGATCCATGGGGAGGAGTCCA | TCTAGATGGCCATTCCTCAGAGT |
| pVvMYB100-GFP | GGTACCATGGTCTCCTGAAGA | TCTAGAAATATGTCCAAAGGTTG |
| pVvMYB170-GFP | GGATCCATGACTCTGACCACCG | TCTAGAGGGATTATTTTGG |
| pVvMYB191-GFP | GGATCCATGGGAAGACCACCT | GTCGACGAAAAAATCAGGACTTT |
| pVvMYB205-GFP | GGATCCATGGGCAGAGCACCT | TCTAGAAATGAGTAGTGATTCGG |
| pVvMYB251-GFP | GGATCCATGGAAGCAACCAAC | TCTAGAACAGTCATGAAACCCAG |
| pVvMYB258-GFP | GGATCCATGGGAAGAACACCA | GTCGACCATAATTGCATTAACAT |
| pVvMYB263-GFP | GGATCCATGGGGAGAG GCAG | TCTAGAAAAGACAAAATTGTGAG |
