# Supplementary figures and images for: Identification of Vitis vinifera MYB transcription factors and their response against grapevine berry inner necrosis virus
Source: BMC Plant Biol. 2023 May 26;23:279. doi: 10.1186/s12870-023-04296-7 (PMC10214588; doi:10.1186/s12870-023-04296-7)

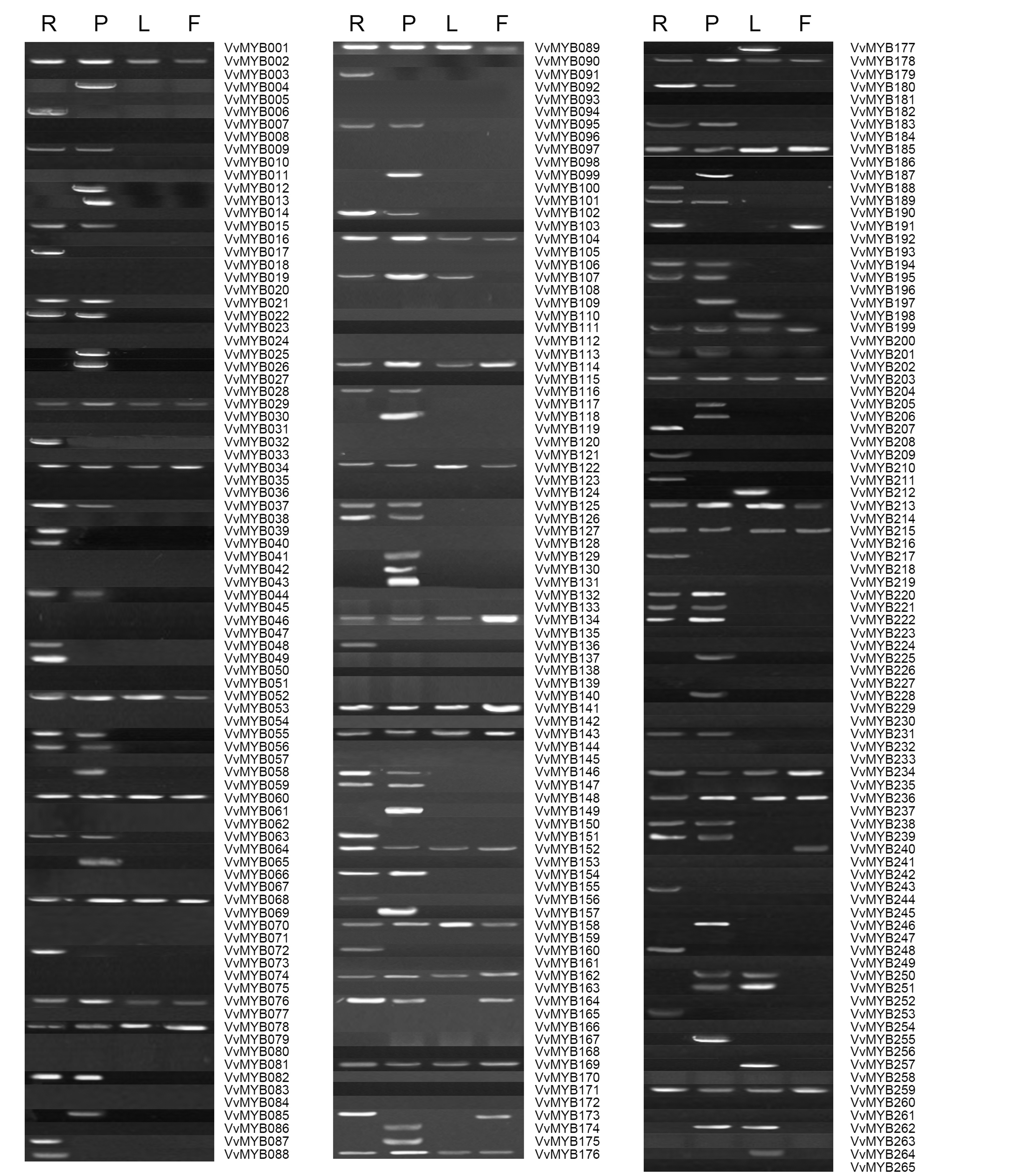

Supplement: Supplementary file 7 — Additional file 7: Fig. S1. Tissue-specific expression patterns of VvMYB genes in “Crimson seedless” grapevine. R, roots; P, phloem; L, leaf blades; F, fruit. [file 12870_2023_4296_MOESM7_ESM.jpg]

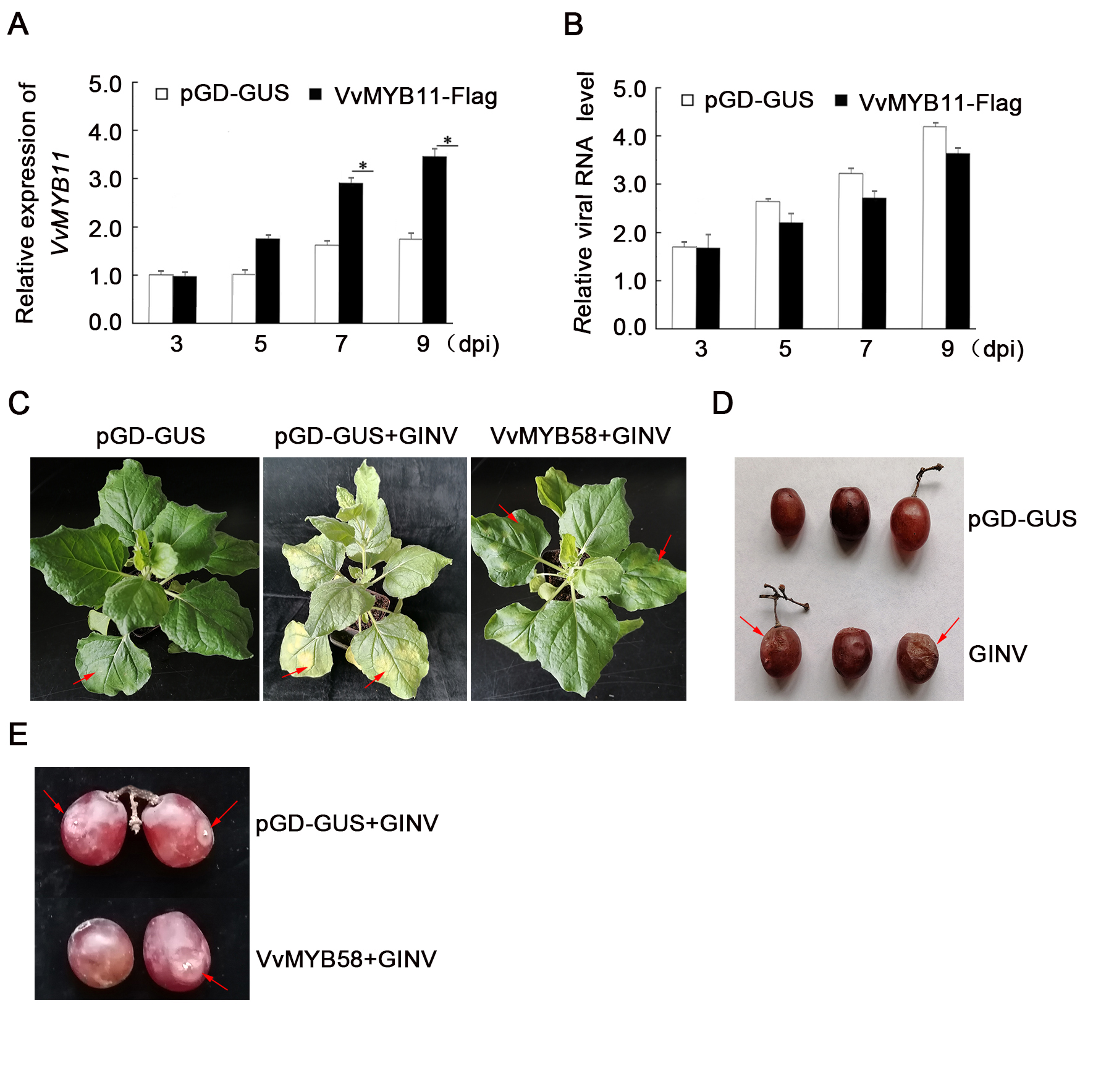

Supplement: Supplementary file 8 — Additional file 8: Fig. S2. Overexpression of VvMYB11 did not affect viral replication. (A) and (B) pGD-VvMYB11-Flag and GINV-GFP were co-infiltrated into grapevine berries by Agrobacterium-mediated transient transformation; pGD-GUS and GINV-GFP served as the negative control. Error bars represent the SD of three independent biological replicates. *P < 0.05, **P < 0.01. Overexpression of VvMYB58 did not affect the symptoms of virus-infected. Comparison of symptoms of GINV virus-infected GINV virus-infected Nicotiana benthamiana (C) and “Crimson seedless” grapevine fruits with mock at 7 dpi (E). (D) shows symptoms of solitary infection GINV of “Crimson seedless” grapevine fruits. [file 12870_2023_4296_MOESM8_ESM.jpg]

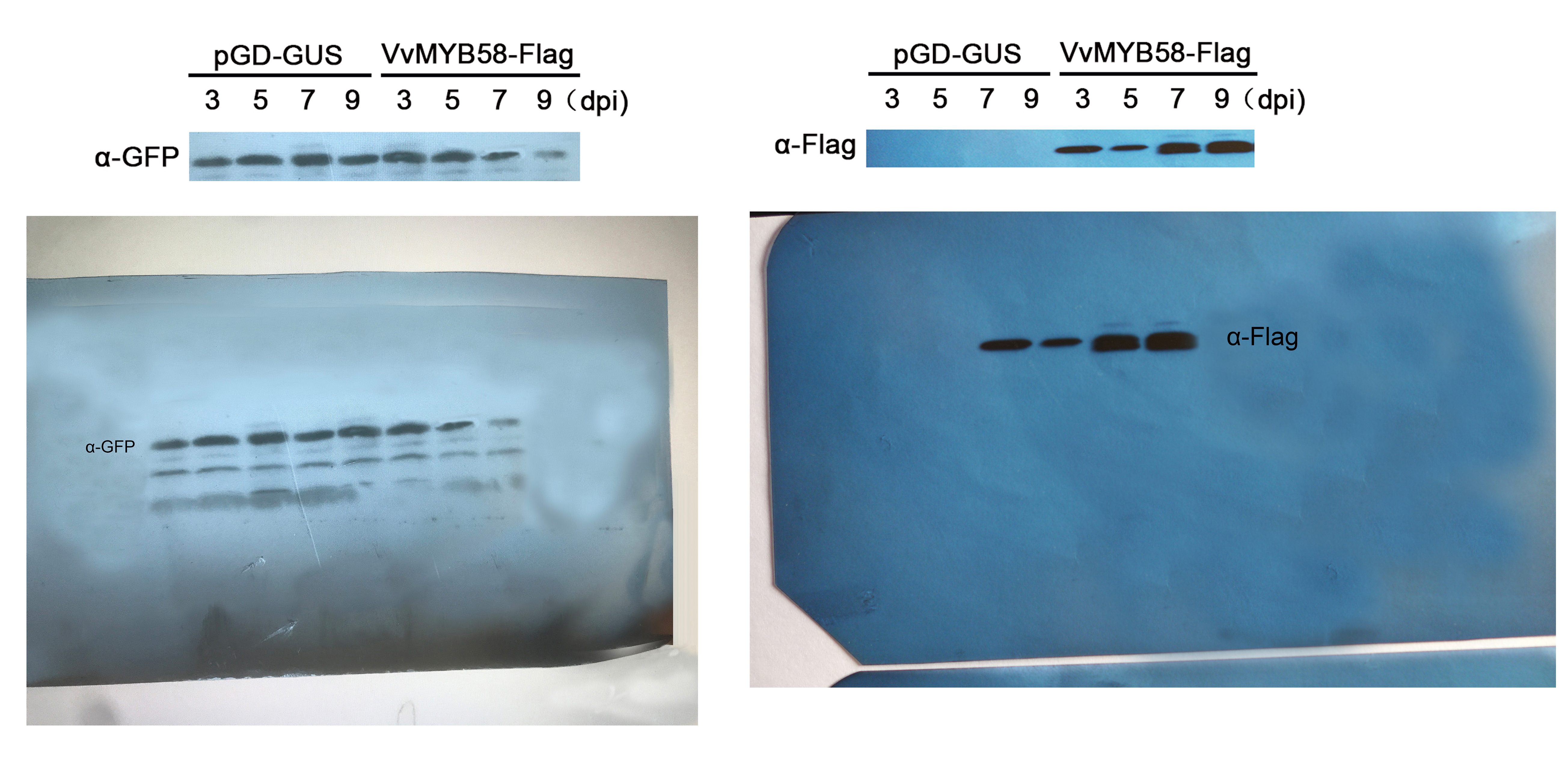

Supplement: Supplementary file 9 — Additional file 9: Fig. S3. The original image blots in Fig. 6C. [file 12870_2023_4296_MOESM9_ESM.jpg]
