# Supplementary material for: Identification of Vitis vinifera MYB transcription factors and their response against grapevine berry inner necrosis virus
Source: BMC Plant Biol. 2023 May 26;23:279. doi: 10.1186/s12870-023-04296-7 (PMC10214588; doi:10.1186/s12870-023-04296-7)
Supplement: Supplementary file 10 — Additional file 10: Fig. S4. Part of the original image of Fig. 3. [file 12870_2023_4296_MOESM10_ESM.pdf]

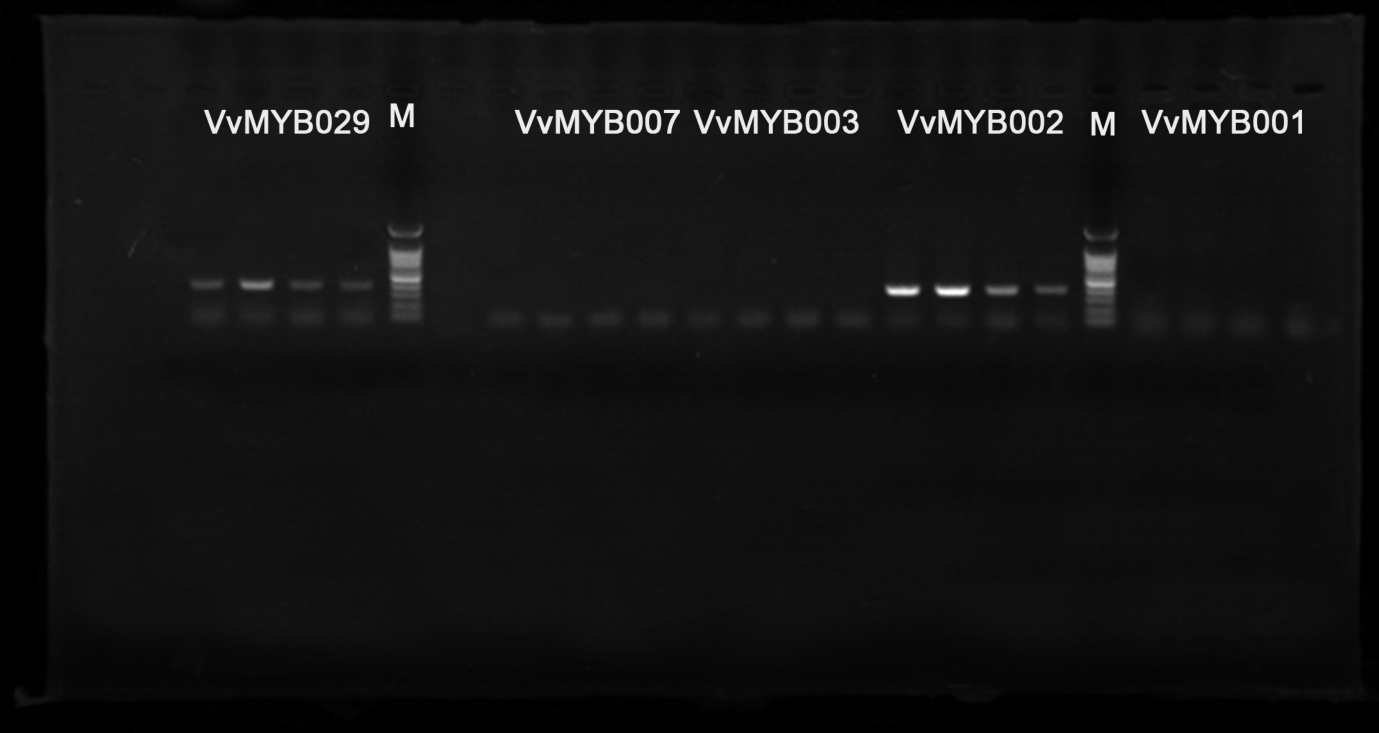
VvMYB029
M
VvMYB007
VvMYB003
VvMYB002
M
VvMYB001

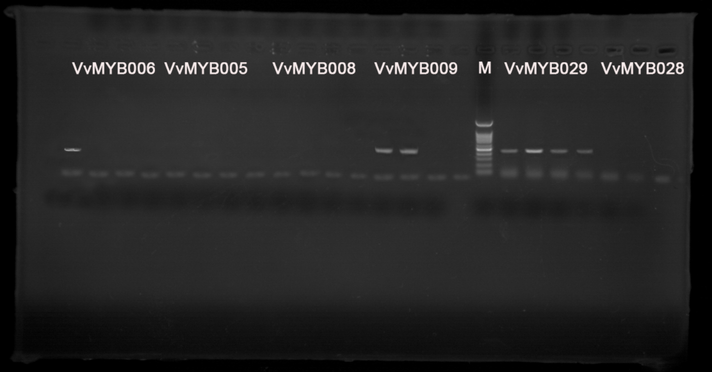
VvMYB006 VvMYB005 VvMYB008 VvMYB009 M VvMYB029 VvMYB028

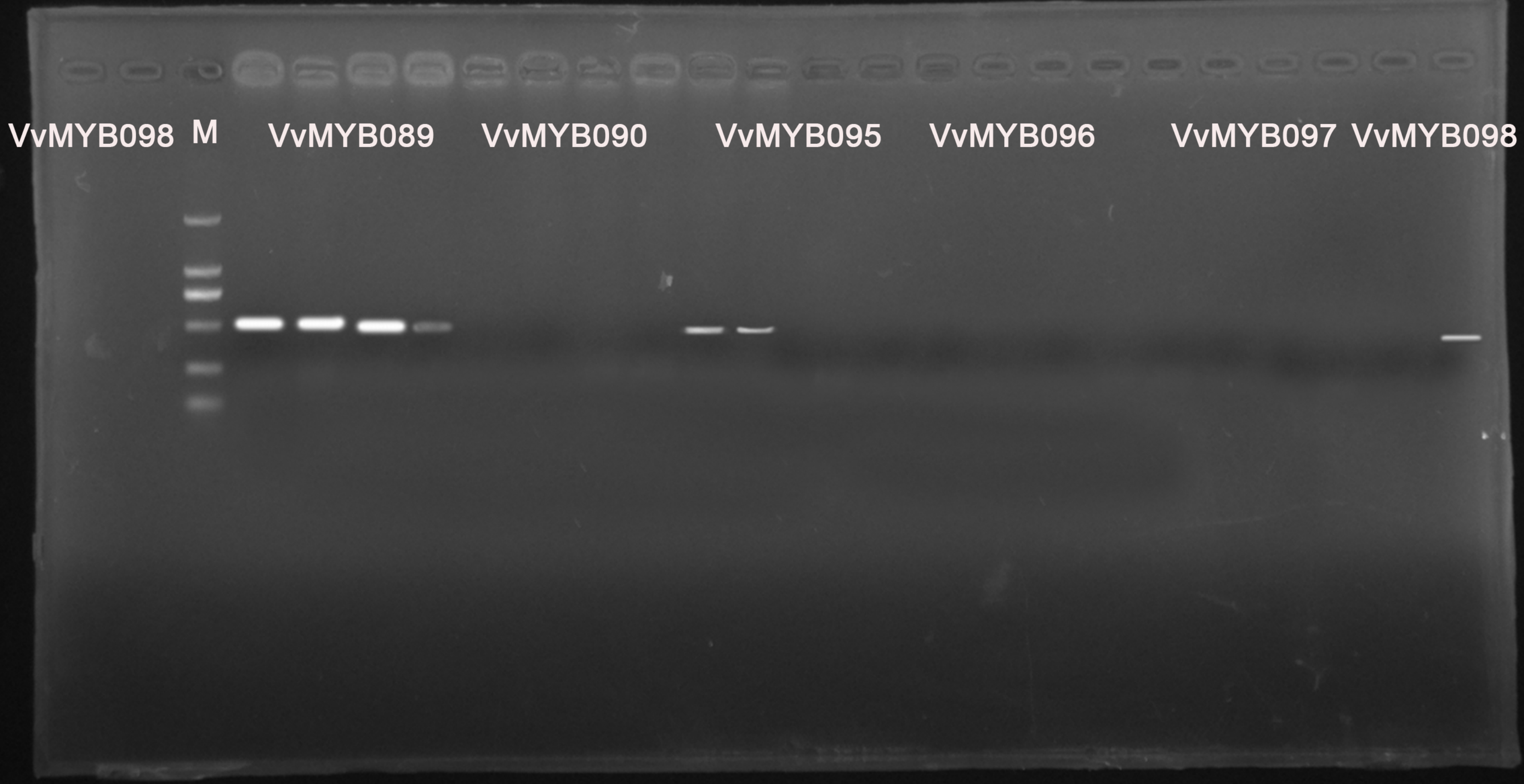
VvMYB098
M
VvMYB089
VvMYB090
VvMYB095
VvMYB096
VvMYB097
VvMYB098

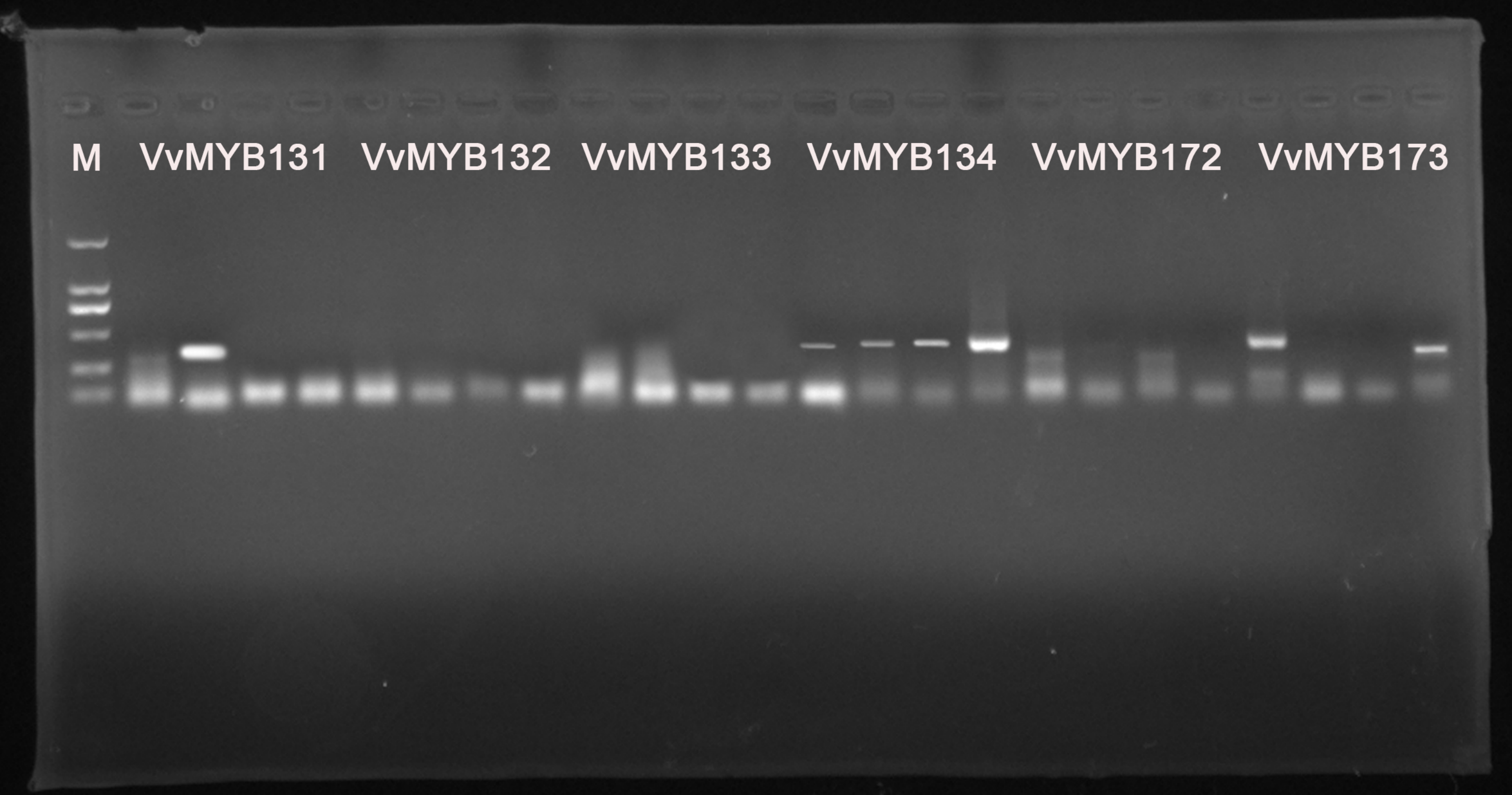
M
VvMYB131
VvMYB132
VvMYB133
VvMYB134
VvMYB172
VvMYB173

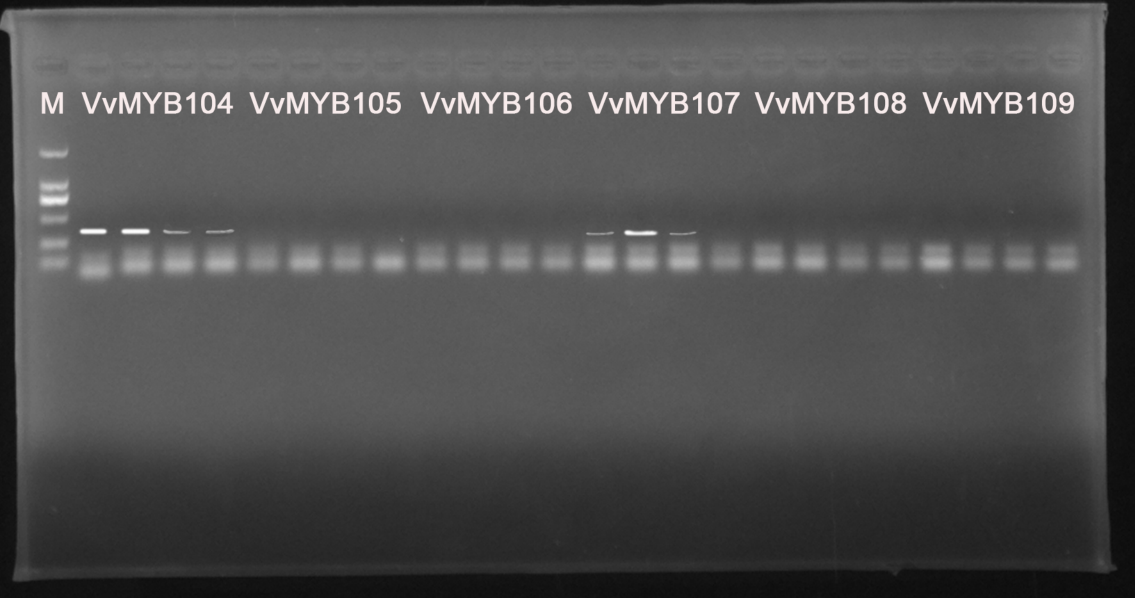
M
VvMYB104
VvMYB105
VvMYB106
VvMYB107
VvMYB108
VvMYB109

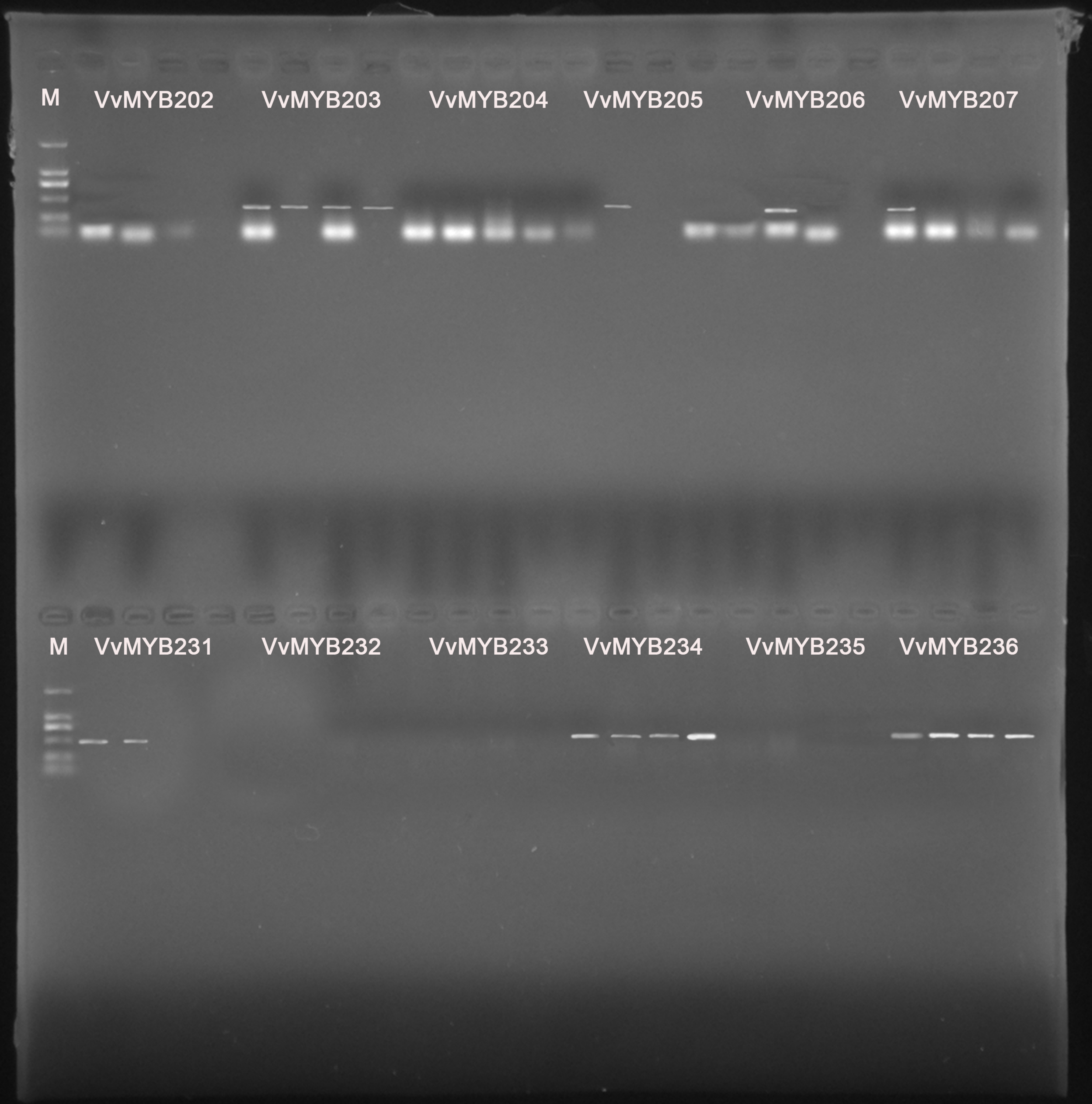
M
VvMYB202
VvMYB203
VvMYB204
VvMYB205
VvMYB206
VvMYB207
M
VvMYB231
VvMYB232
VvMYB233
VvMYB234
VvMYB235
VvMYB236

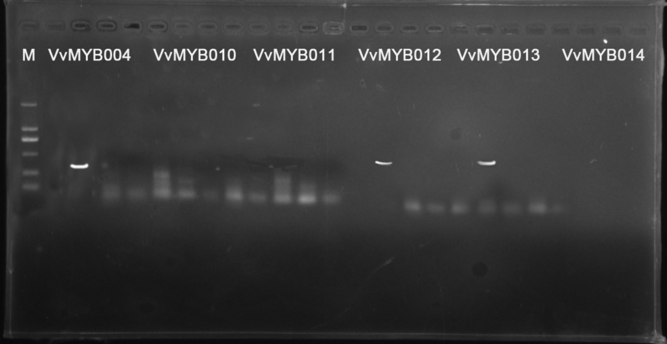
M
VvMYB004
VvMYB010
VvMYB011
VvMYB012
VvMYB013
VvMYB014

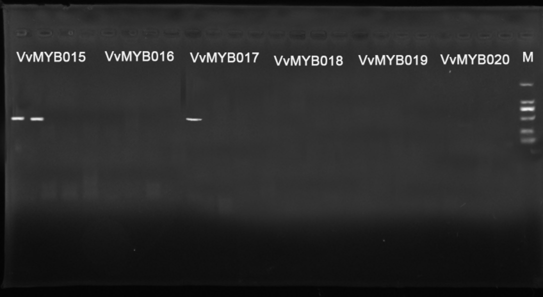
VvMYB015
VvMYB016
VvMYB017
VvMYB018
VvMYB019
VvMYB020
M

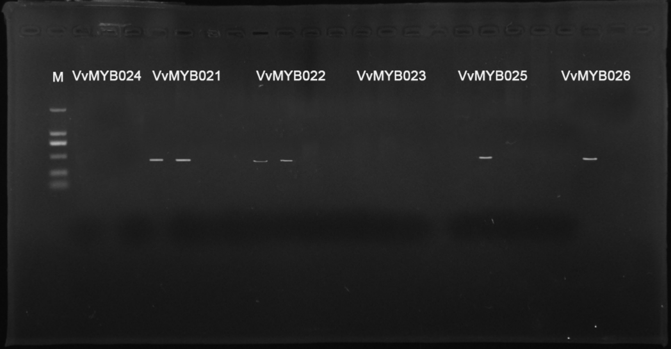

M
VvMYB024
VvMYB021
VvMYB022
VvMYB023
VvMYB025
VvMYB026

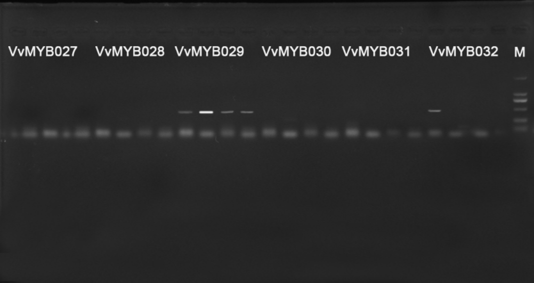
VvMYB027
VvMYB028
VvMYB029
VvMYB030
VvMYB031
VvMYB032
M

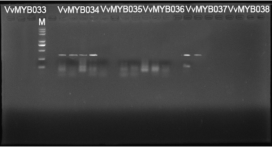
VvMYB033
VvMYB034
VvMYB035
VvMYB036
VvMYB037
VvMYB038
M

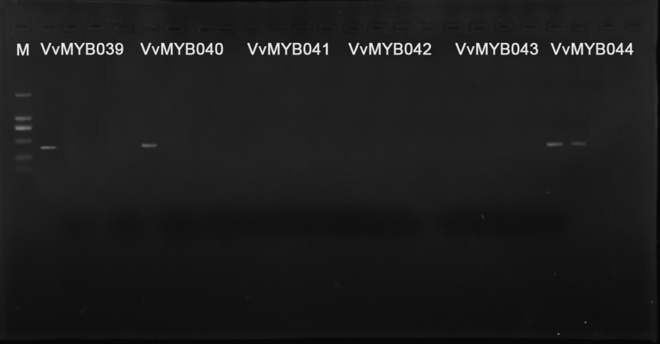
M
VvMYB039
VvMYB040
VvMYB041
VvMYB042
VvMYB043
VvMYB044

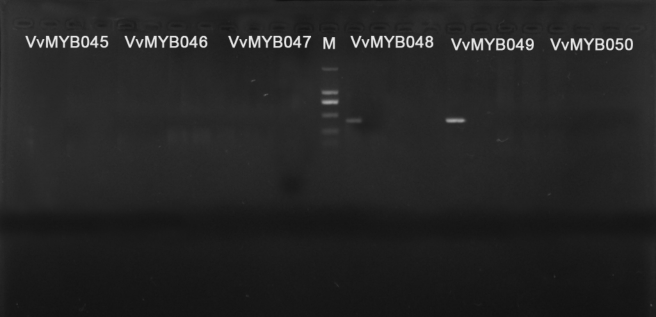
VvMYB045
VvMYB046
VvMYB047
M
VvMYB048
VvMYB049
VvMYB050

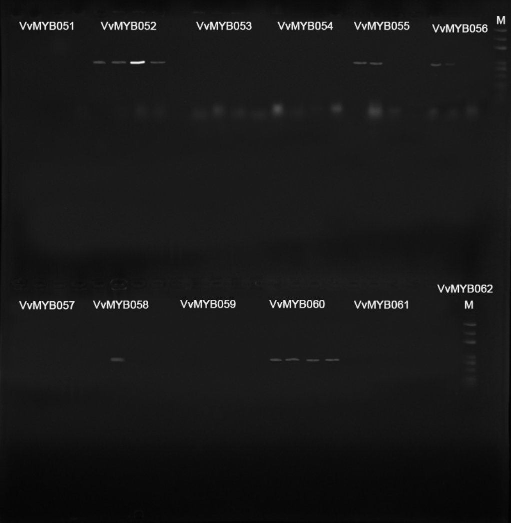
VvMYB051
VvMYB052
VvMYB053
VvMYB054
VvMYB055
VvMYB056
M
VvMYB057
VvMYB058
VvMYB059
VvMYB060
VvMYB061
VvMYB062
M

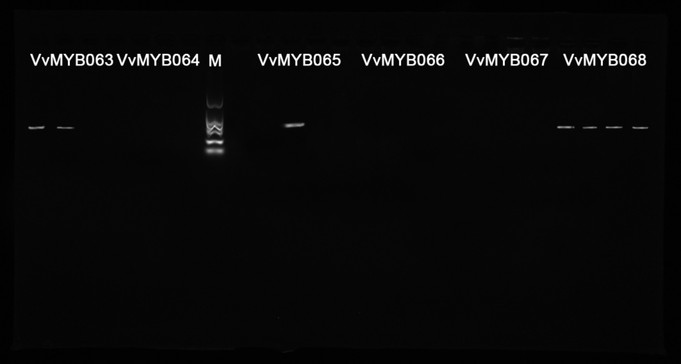
VvMYB063
VvMYB064
M
VvMYB065
VvMYB066
VvMYB067
VvMYB068

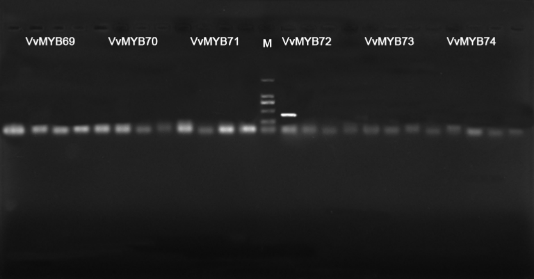
VvMYB69
VvMYB70
VvMYB71
M
VvMYB72
VvMYB73
VvMYB74

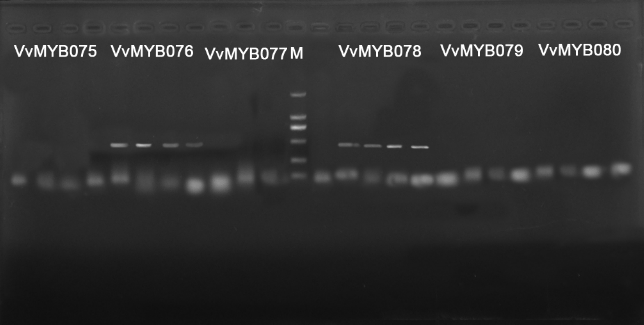
VvMYB075
VvMYB076
VvMYB077
M
VvMYB078
VvMYB079
VvMYB080

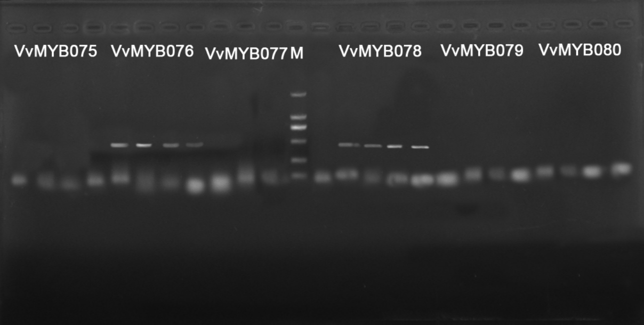
VvMYB075
VvMYB076
VvMYB077
M
VvMYB078
VvMYB079
VvMYB080

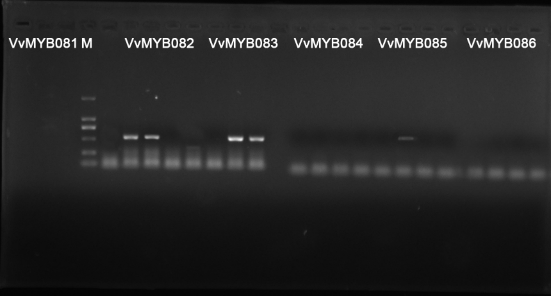
VvMYB081 M
VvMYB082
VvMYB083
VvMYB084
VvMYB085
VvMYB086

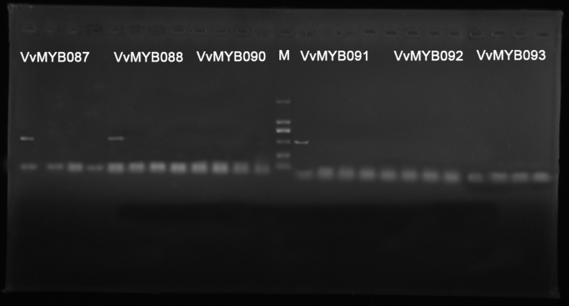
VvMYB087
VvMYB088
VvMYB090
M
VvMYB091
VvMYB092
VvMYB093

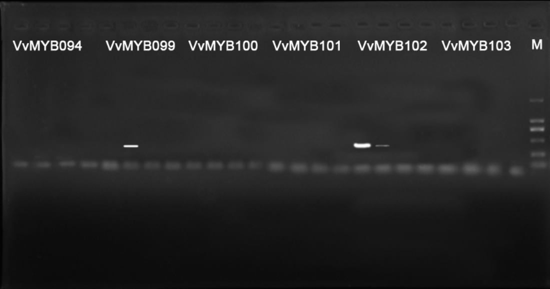
VvMYB094
VvMYB099
VvMYB100
VvMYB101
VvMYB102
VvMYB103
M

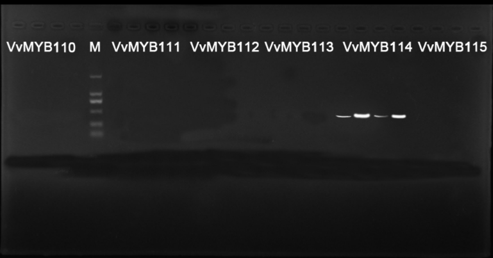
VvMYB110
M
VvMYB111
VvMYB112
VvMYB113
VvMYB114
VvMYB115

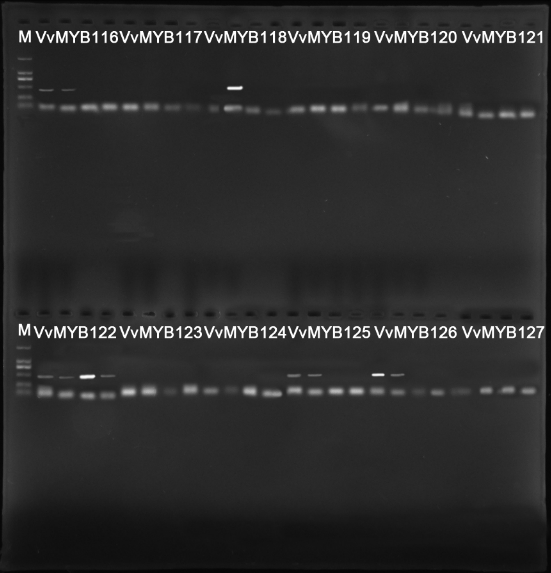

M
VvMYB116
VvMYB117
VvMYB118
VvMYB119
VvMYB120
VvMYB121
M
VvMYB122
VvMYB123
VvMYB124
VvMYB125
VvMYB126
VvMYB127

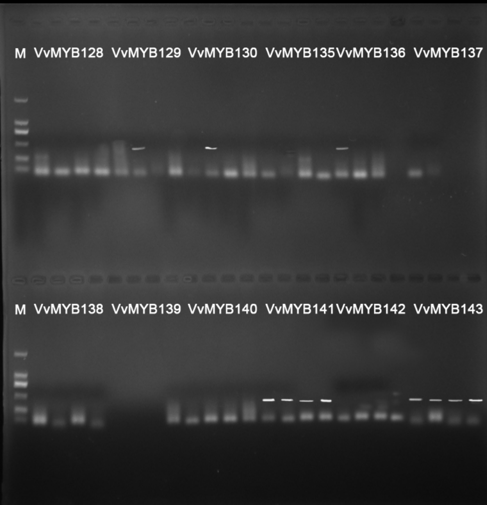
M
VvMYB128
VvMYB129
VvMYB130
VvMYB135
VvMYB136
VvMYB137
M
VvMYB138
VvMYB139
VvMYB140
VvMYB141
VvMYB142
VvMYB143

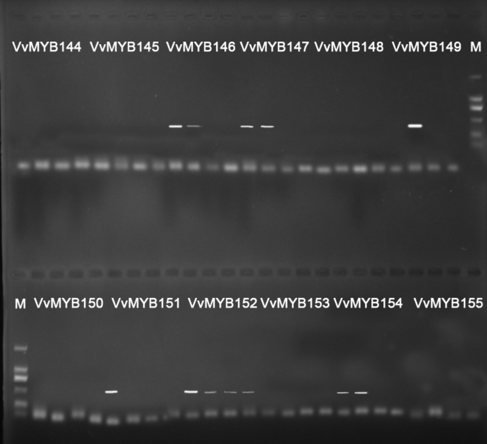
VvMYB144 VvMYB145 VvMYB146 VvMYB147 VvMYB148 VvMYB149 M
M VvMYB150 VvMYB151 VvMYB152 VvMYB153 VvMYB154 VvMYB155

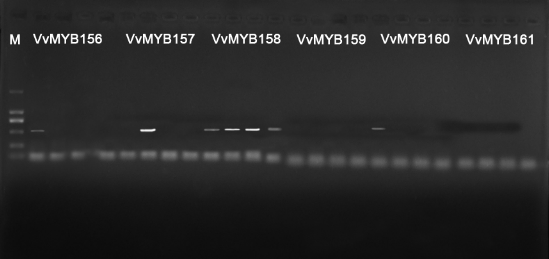
M
VvMYB156
VvMYB157
VvMYB158
VvMYB159
VvMYB160
VvMYB161

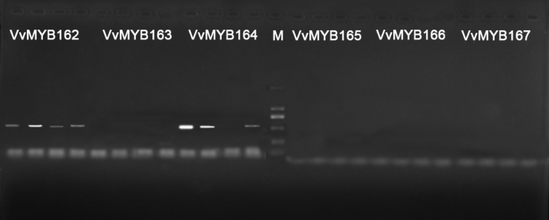

VvMYB162
VvMYB163
VvMYB164
M
VvMYB165
VvMYB166
VvMYB167

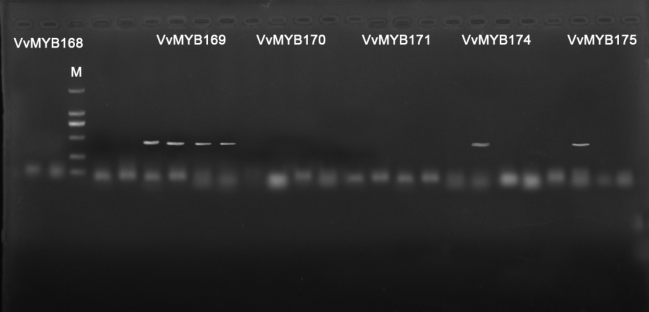

VvMYB168
M
VvMYB169
VvMYB170
VvMYB171
VvMYB174
VvMYB175

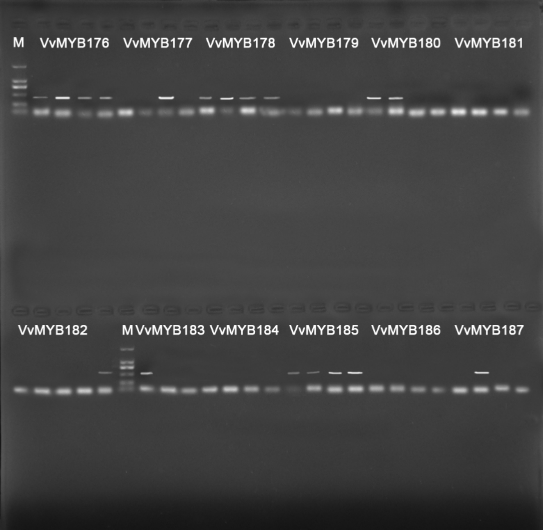
M
VvMYB176
VvMYB177
VvMYB178
VvMYB179
VvMYB180
VvMYB181
VvMYB182
M
VvMYB183
VvMYB184
VvMYB185
VvMYB186
VvMYB187

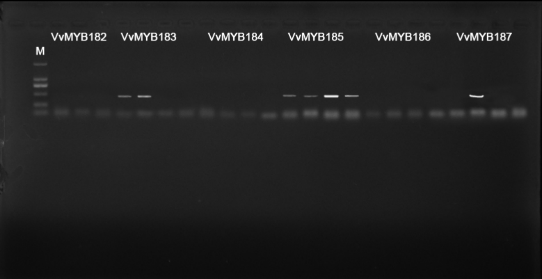
VvMYB182
VvMYB183
VvMYB184
VvMYB185
VvMYB186
VvMYB187
M

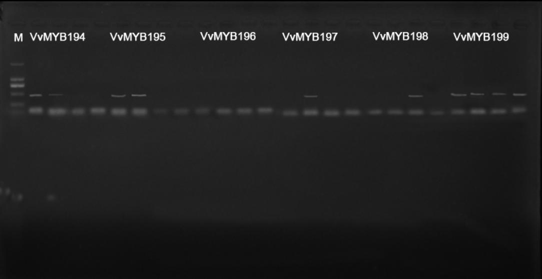
M
VvMYB194
VvMYB195
VvMYB196
VvMYB197
VvMYB198
VvMYB199

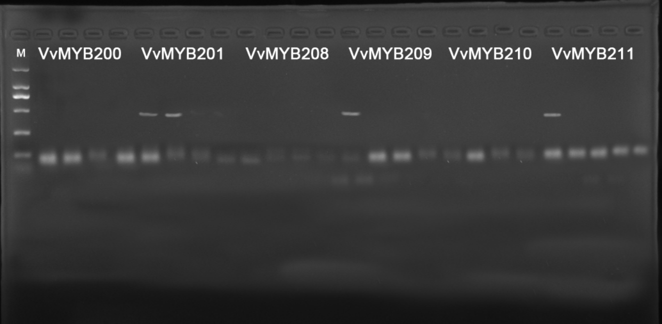
M
VvMYB200
VvMYB201
VvMYB208
VvMYB209
VvMYB210
VvMYB211

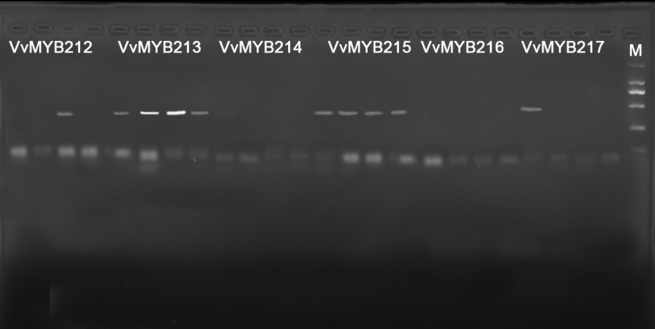
VvMYB212
VvMYB213
VvMYB214
VvMYB215
VvMYB216
VvMYB217
M

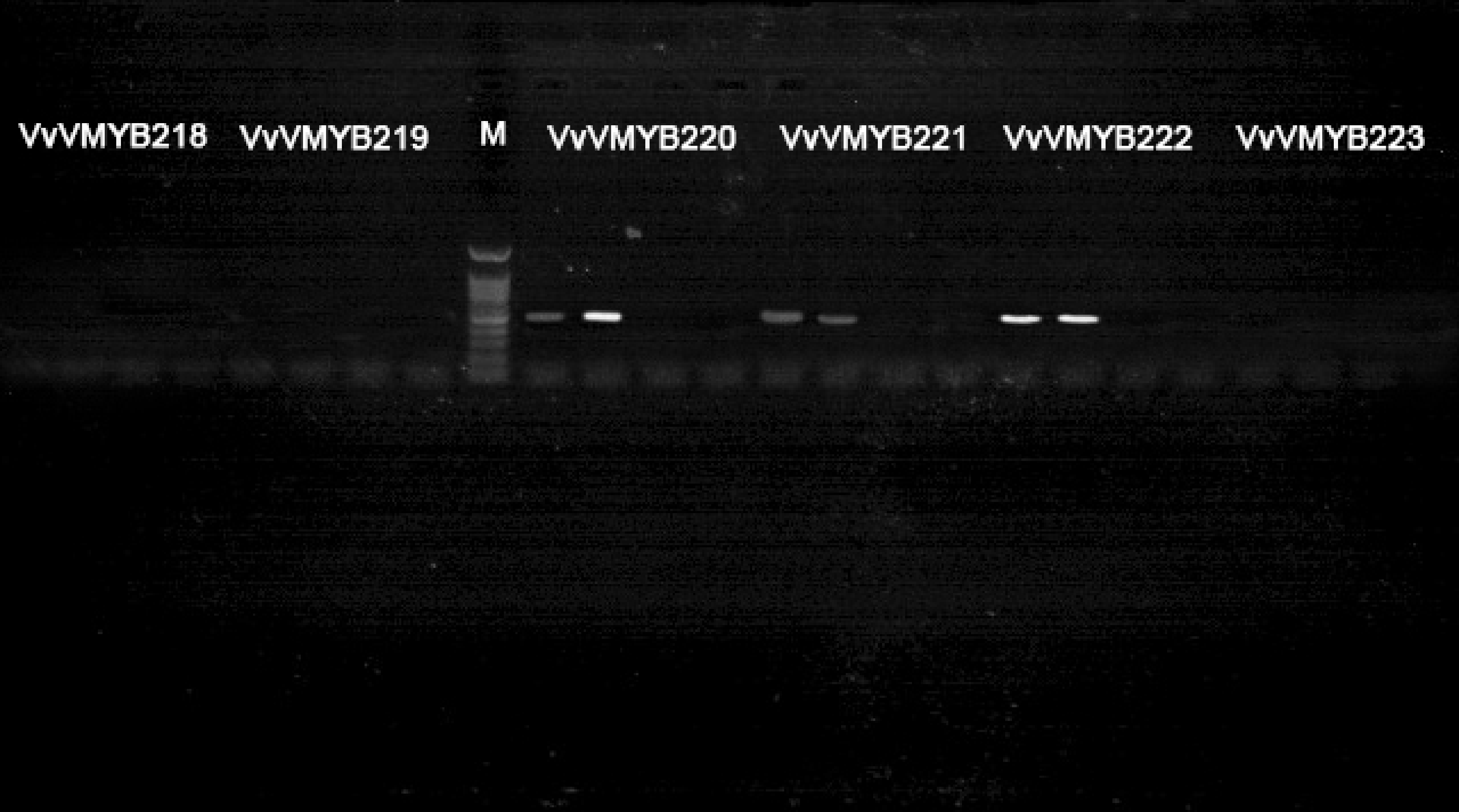
VvVMYB218
VvVMYB219
M
VvVMYB220
VvVMYB221
VvVMYB222
VvVMYB223

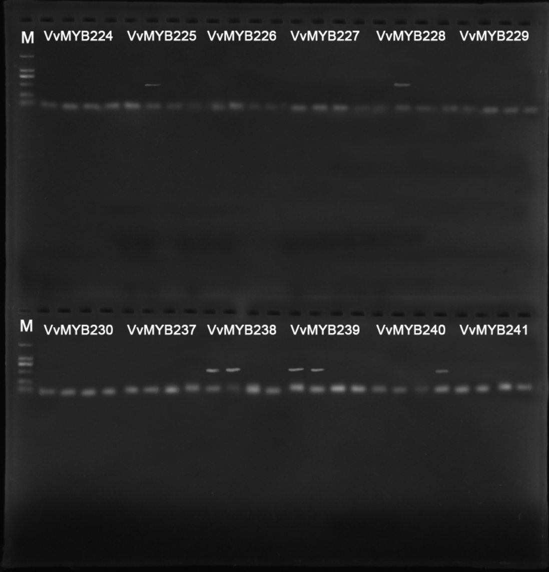
M
VvMYB224
VvMYB225
VvMYB226
VvMYB227
VvMYB228
VvMYB229
M
VvMYB230
VvMYB237
VvMYB238
VvMYB239
VvMYB240
VvMYB241

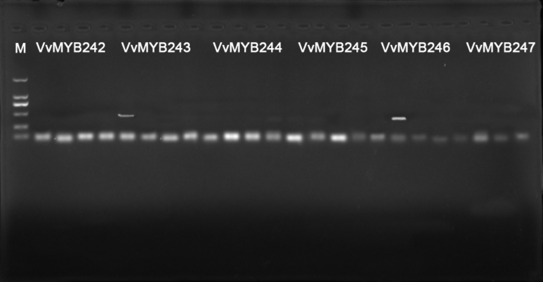
M
VvMYB242
VvMYB243
VvMYB244
VvMYB245
VvMYB246
VvMYB247

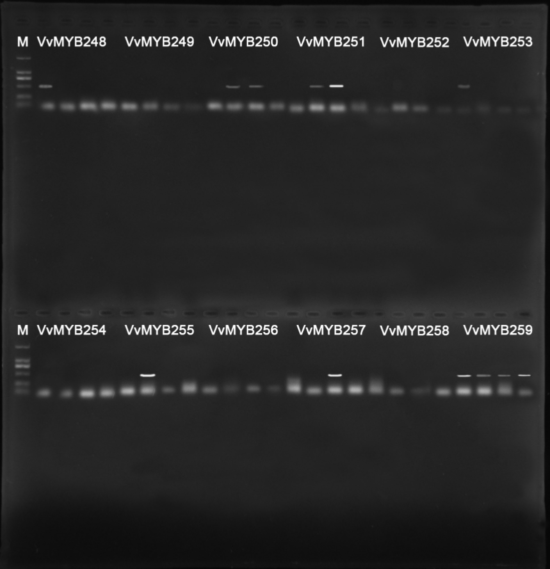
M
VvMYB248
VvMYB249
VvMYB250
VvMYB251
VvMYB252
VvMYB253
M
VvMYB254
VvMYB255
VvMYB256
VvMYB257
VvMYB258
VvMYB259

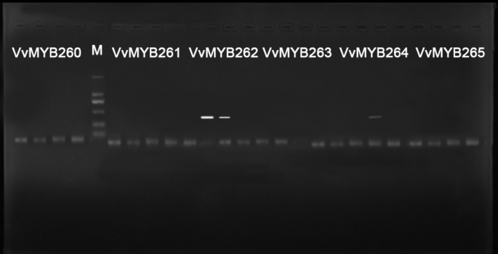
VvMYB260
M
VvMYB261
VvMYB262
VvMYB263
VvMYB264
VvMYB265
